# Ribosomal Stalk Protein Silencing Partially Corrects the ΔF508-CFTR Functional Expression Defect

**DOI:** 10.1371/journal.pbio.1002462

**Published:** 2016-05-11

**Authors:** Guido Veit, Kathryn Oliver, Pirjo M. Apaja, Doranda Perdomo, Aurélien Bidaud-Meynard, Sheng-Ting Lin, Jingyu Guo, Mert Icyuz, Eric J. Sorscher, John L. Hartman, Gergely L. Lukacs

**Affiliations:** 1 Department of Physiology, McGill University, Montréal, Quebec, Canada; 2 Department of Genetics, University of Alabama at Birmingham, Birmingham, Alabama, United States of America; 3 Gregory Fleming James Cystic Fibrosis Research Center, University of Alabama at Birmingham, Birmingham, Alabama, United States of America; 4 Department of Pediatrics, Emory University School of Medicine, Atlanta, Georgia, United States of America; 5 Department of Biochemistry, McGill University, Montréal, Quebec, Canada; 6 Groupe de Recherche Axé sur la Structure des Protéines (GRASP), McGill University, Montréal, Quebec, Canada; University of Cambridge, UNITED KINGDOM

## Abstract

The most common cystic fibrosis (CF) causing mutation, deletion of phenylalanine 508 (ΔF508 or Phe508del), results in functional expression defect of the CF transmembrane conductance regulator (CFTR) at the apical plasma membrane (PM) of secretory epithelia, which is attributed to the degradation of the misfolded channel at the endoplasmic reticulum (ER). Deletion of phenylalanine 670 (ΔF670) in the yeast oligomycin resistance 1 gene (*YOR1*, an ABC transporter) of *Saccharomyces cerevisiae* phenocopies the ΔF508-CFTR folding and trafficking defects. Genome-wide phenotypic (phenomic) analysis of the Yor1-ΔF670 biogenesis identified several modifier genes of mRNA processing and translation, which conferred oligomycin resistance to yeast. Silencing of orthologues of these candidate genes enhanced the ΔF508-CFTR functional expression at the apical PM in human CF bronchial epithelia. Although knockdown of *RPL12*, a component of the ribosomal stalk, attenuated the translational elongation rate, it increased the folding efficiency as well as the conformational stability of the ΔF508-CFTR, manifesting in 3-fold augmented PM density and function of the mutant. Combination of *RPL12* knockdown with the corrector drug, VX-809 (lumacaftor) restored the mutant function to ~50% of the wild-type channel in primary *CFTR*^*ΔF508/ΔF508*^ human bronchial epithelia. These results and the observation that silencing of other ribosomal stalk proteins partially rescue the loss-of-function phenotype of ΔF508-CFTR suggest that the ribosomal stalk modulates the folding efficiency of the mutant and is a potential therapeutic target for correction of the ΔF508-CFTR folding defect.

## Abstract 2

Reducing the rate of translational elongation by silencing ribosomal stalk proteins ameliorates the folding and stability defect of the cystic fibrosis mutant protein ΔF508-CFTR, partially restoring the plasma membrane chloride conductance.

## Author Summary

Cystic fibrosis (CF) is one of the most common autosomal recessive diseases in Caucasians. It is caused by mutations in the CF transmembrane conductance regulator (CFTR), which functions as an anion channel at the apical plasma membrane of secretory epithelia. The most common CF mutation, a deletion of the phenylalanine residue at position 508 (ΔF508), results in the channel misfolding and subsequent intracellular degradation. Our previous genome-wide phenotypic screens, using a yeast variant, have predicted modifier genes for ΔF508-CFTR biogenesis. Here, we show that silencing of one of these candidate genes, *RPL12*, a component of the ribosomal stalk, increased the folding and stabilization of ΔF508-CFTR, resulting in its increased plasma membrane expression and function. Our data suggest that reducing the translational elongation rate via *RPL12* silencing can partially reverse the ΔF508-CFTR folding defect. Importantly, *RPL12* silencing in combination with the corrector drug VX-809 (lumacaftor), increased the mutant function to 50% of the wild-type CFTR channel, suggesting that the ribosomal stalk perturbation may represent a therapeutic target for rescuing the ΔF508-CFTR biogenesis defect.

## Introduction

Cystic fibrosis (CF), caused by mutations in cystic fibrosis transmembrane conductance regulator (CFTR), is characterized by multiorgan pathology, mainly affecting the upper and lower airways, gastrointestinal tract, and endocrine system [1,2]. To date ~2,000 mutations have been identified in the *CFTR* gene with widely variable disease severity [3–5]. The gene product, CFTR, is an ATP-binding cassette (ABC) transporter, which functions as a cyclic AMP-regulated chloride and bicarbonate channel in secretory epithelia [2,6]. Deletion of the phenylalanine at position 508 (Phe508del, designated as ΔF508) in the nucleotide binding domain 1 (NBD1), the most common CF-causing mutation, results in misfolding and premature degradation of the mutant via the endoplasmic reticulum (ER)-associated degradation pathway (ERAD) [7–9]. The small amount of ΔF508-CFTR molecules that escape the ER are functionally, conformationally, and biochemically unstable and are rapidly removed from the plasma membrane (PM) via the endolysosomal associated degradation pathway [10,11].

To rescue the folding defect of ΔF508-CFTR, several strategies have been pursued with limited success so far [12–14]. Small molecule correctors that act as pharmacological chaperones, like VX-809, can directly bind to and promote the folding of ΔF508-CFTR [15–19]. In combination with the gating potentiator VX-770, VX-809 achieved only modest benefit in CF patients homozygous for the ΔF508 mutation [20], which might be attributed in part to the destabilization of ΔF508-CFTR upon chronic exposure to VX-770 [21,22]. Modifier genes may also facilitate the ΔF508-CFTR functional rescue by enhancing the mRNA or protein expression, folding, stability, or by inhibiting its degradation at the ER and post-ER compartments [11,12, 23–25]. Candidate modifier genes have been isolated by genome-wide SNP studies [26,27], identification of the CFTR interactome [28–30], and phenotypic screens of targted siRNA libraries [11,31]. As a complementary approach, strategies focusing on reverting the maladaptive stress response in CF have been proposed [32]. None of these approaches, however, appear to attain sufficient functional correction in preclinical studies to be therapeutically robust in patients with the most common CF mutation, particularly in individuals carrying only one copy of ΔF508-CFTR, representing 40% of US CF patients [3].

Chimeras between the yeast ABC transporter STE6 and ΔF508-CFTR were used as a homology model to identify revertant mutations [33], but these chimeras are not recognized by the ER quality control [34]. Recently, we have employed a genome-wide screen to identify modifiers of CFTR misfolding, utilizing high-throughput yeast phenomic analysis of Yor1, a member of the ABC transporter superfamily, with deletion of phenylalanine 670 (Yor1-ΔF670). The ΔF508-equivalent mutation, Yor1-ΔF670, results in protein misfolding, ER retention, and proteasomal degradation similar to that of ΔF508-CFTR in mammalian cells [35–37]. Oligomycin, which inhibits the ATP synthase, is extruded by Yor1 across the PM, enabling a screen of the yeast gene deletion strain library [37,38] for modulators of Yor1-ΔF670 processing as determined by oligomycin sensitivity. Our phenomic screen provided a comprehensive gene interaction network that can potentially modulate ΔF508-CFTR biogenesis [39]. Evolutionary conservation in the ΔF-biogenesis network was demonstrated by the identification of many yeast homologs of published human genes that modulate ΔF508-CFTR biogenesis similarly to that of Yor1-ΔF670 function [39].

Here, we validated a subset of genes that were identified by quantitative high throughput cell array phenotyping (Q-HTCP) in the yeast model, including components of the cytoplasmic exosome, rRNA biogenesis pathway, and most notably, the ribosomal stalk, in human respiratory cells. We show that silencing of ribosomal stalk proteins, in particular *RPL12*, increases the rescued ΔF508-CFTR PM density, function, and thermal stability, suggesting that ribosomal stalk proteins have a determinant role in the folding of the mutant channel and may represent a possible therapeutic target for correction of the ΔF508-CFTR folding defect. Furthermore, the results highlight the capacity of yeast phenomic screen as a systematic approach to investigate disease modifier genes, adding CF to a list of human diseases where yeast can function as a powerful model system [40–42].

## Results

### Phenomic Analysis Reveals Evolutionarily Conserved Gene Interaction Modules That Influence Yor1-ΔF670 Function

**Fig 1:**
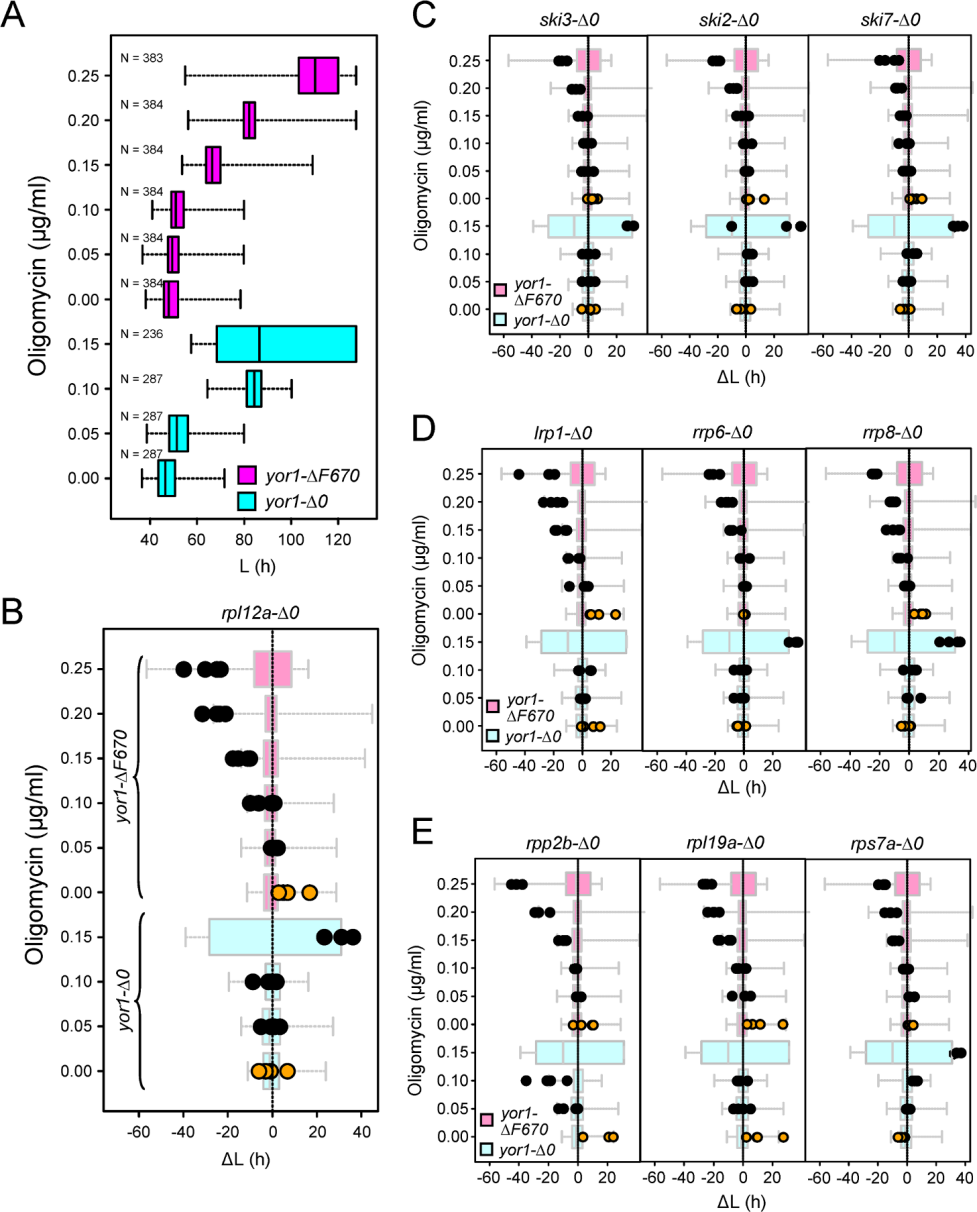
Genes involved in the function of the cytoplasmic exosome, nuclear exosome, rRNA processing and ribosome influence the function of Yor1-ΔF670. **(A)** Box-whisker plot of the cell proliferation parameter L (time to reach half-maximal carrying capacity) of the *yor1-Δ0* (gene deletion) strain (cyan) and the *yor1-ΔF670* mutant (magenta) following treatment with oligomycin at multiple concentrations. 287 cultures were analyzed for *yor1-Δ0* and 384 cultures for *yor1-ΔF670*. “N < 287” or “N < 384” indicates that the concentration of oligomycin was fully growth inhibitory for some of the replicate cultures. Box plots indicate the central 75% of values, whiskers the total value range and averages are indicated by black bars. At higher growth inhibitory concentrations, the phenotypic distributions widen, and thus the high range for the *yor1-Δ0* at 0.15 ug/mL oligomycin is not depicted to permit better visualization of the data. The single mutant reference strain data from panel A is dose-normalized and plotted in the background for the identically normalized double-mutant data (black dots) in panels B–E to illustrate how each gene deletion influences the oligomycin resistance associated with the *yor1-Δ0* and *yor1-ΔF* alleles. **(B)** The *RPL12A* deletion increases oligomycin resistance in the context of *yor1-ΔF670* (magenta), but not *yor1-Δ0* (cyan). Oligomycin resistance was compared between the single mutants (shown in panel A) and the respective double mutant cultures, separately constructed in quadruplicate (biological replicates, black circles). Data for each series of replicates was normalized by the difference in L in the absence of oligomcyin (orange circles). **(C–E)** Plots similar to those in panel B are shown for gene modules comprising the cytoplasmic exosome involved in mRNA regulation (C), genes functioning in the nuclear exosome and rRNA processing (D), and additional ribosomal proteins (E). See **Table 1** for additional information. The underlying data of panels A–E can be found in S1 Data.

To select hits from our previous yeast screen [39] for further study in human cells, the top 180 gene deletions that increased oligomycin resistance of Yor1-ΔF670-associated oligomycin sensitivity (or deletion suppressors) were prioritized by retesting and secondary screening with additional experimental controls (described below). The hits were also subjected to analysis by the DAVID bioinformatics tool [43]. Functional annotation clustering identified two groups, ribosomal or ribosome-associated genes and genes involved in RNA degradation, with an enrichment score of > 2 (**S1A Fig**). These analyses revealed several gene interaction modules (e.g. multiple subunits of a protein complex) and, when analyzed in parallel with the *yor1-Δ0* (null) allele, verified that oligomycin resistance required interaction with Yor1-ΔF670 (**Fig 1**). Additionally, Yor1-ΔF670 interactions were confirmed by expressing the protein from a different promoter and without the C-terminal GFP fusion, that was used in our original screen. Double mutants were remade in quadruplicate using modified synthetic genetic array (SGA) methodology [44,45], and the new panel of double mutants was subjected to Q-HTCP to obtain growth curves at multiple oligomycin concentrations [37]. Growth curves were fit to a logistic growth function, and gene interaction was measured with respect to the cell proliferation parameter, L (**Fig 1A**), which corresponds to the time at which a culture reaches half of the carrying capacity [46]. Multiple components of the cytoplasmic exosome (*SKI2*, *SKI3*, *SKI7)* (**Figs 1C and S1B**), genes involved in ribosomal RNA processing and the nuclear exosome (*LRP1*, *RRP6*, *and RRP8*) (**Figs 1D and S1B**), and ribosomal structural proteins (*RPL12A*, *RPP2B*, *RPS7A*, *and RPL19A*) (**Figs 1B, 1E and S1B**) were identified based on increased resistance to oligomycin upon the gene deletion, in the context of Yor1-ΔF670, but not *yor1-Δ0* (**Fig 1**). On this basis, we focused on the cytoplasmic and nuclear exosome, ribosomal RNA biogenesis, and ribosomal proteins in order to test for the evolutionarily conserved influence of their knockdown on ΔF508-CFTR biogenesis in mammalian cells (**Table 1**).

**Table 1:** Yeast deletion suppressors of Yor1-ΔF670 grouped by functional module with human homologs and functional annotations.

Yeast gene	Human gene	Human UniProt ID	Yeast functional annotation
*SKI2*	*SKIV2L*	Q15477	Ski complex component and putative RNA helicase; mediates 3'→5' RNA degradation by the cytoplasmic exosome
*SKI3*	*TTC37*	Q6PGP7	Ski complex component and TPR protein; mediates 3'→5' RNA degradation by the cytoplasmic exosome
*SKI7*			Coupling protein that mediates interactions between the Ski complex and the cytoplasmic exosome
*LRP1*			Nuclear exosome-associated nucleic acid binding protein
*RRP6*	*EXOSC10*	Q01780	Nuclear exosome exonuclease component
*RRP8*	*HUCE1*	O43159	Nucleolar S-adenosylmethionine-dependent rRNA methyltransferase
*RPL12A*	*RPL12*	P30050	Component of 60S ribosomal subunit stalk
*RPP2B*	*RPLP2*	P05387	Component of 60S ribosomal subunit stalk
*RPL19A*	*RPL19*	P84098	Component of 60S ribosomal subunit
*RPS7A*	*RPS7*	P62081	Component of 40S ribosomal subunit

### Silencing Human Homologs of Yeast Yor-1-ΔF670 Biogenesis Modifier Genes Similarly Influences PM Expression of ΔF508-CFTR

**Fig 2:**
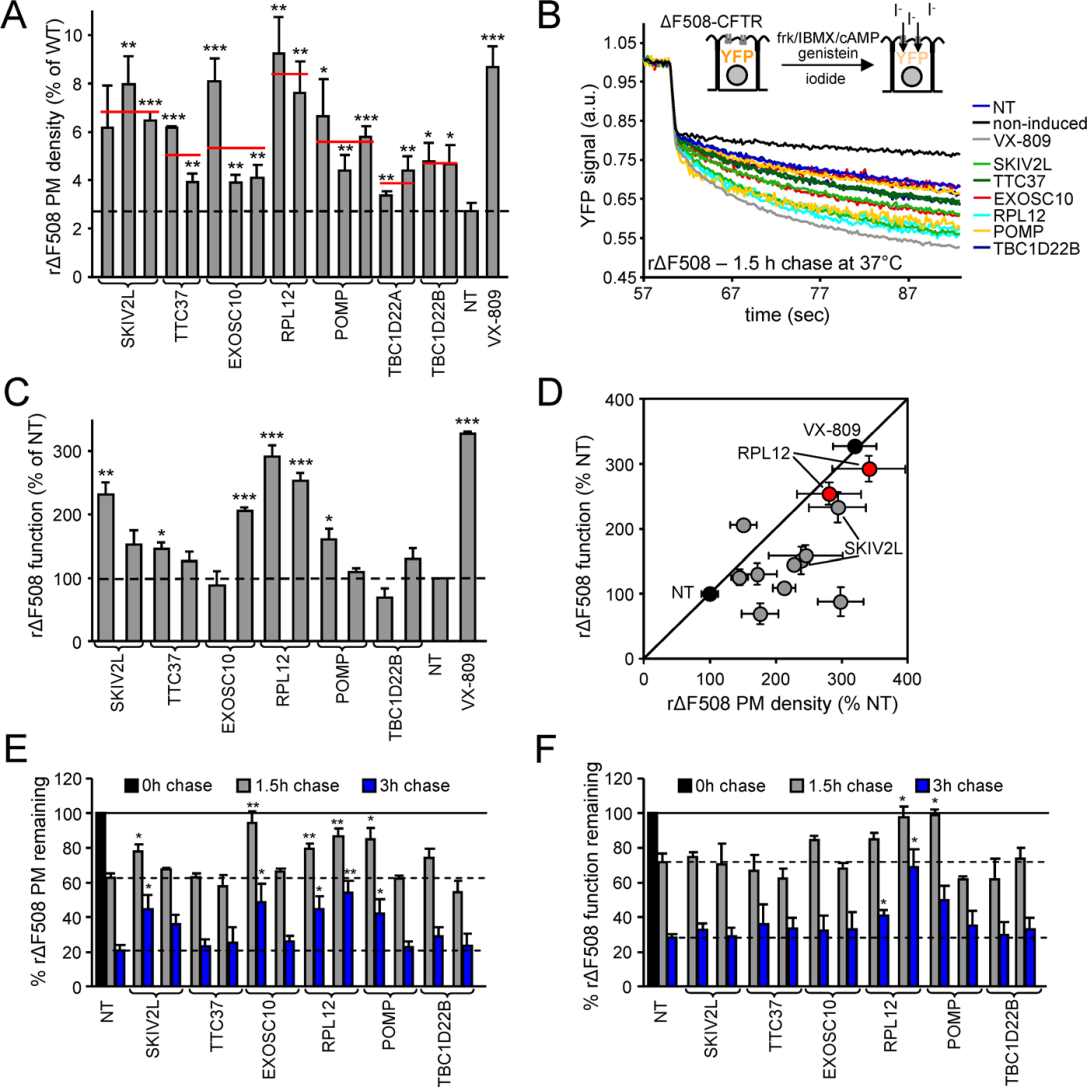
Knockdown of the human homologs of Yor1-ΔF670 modifier genes increases the biochemical and functional expression and stability of the low temperature rescued ΔF508-CFTR. **(A)** PM density of rΔF508-CFTR (48 h, 26°C) after 1 h chase at 37°C was determined by cell surface ELISA in human CFBE, expressing CFTR under the control of tetracycline inducible transactivator and is expressed as percent of wild-type (WT) CFTR. Indicated genes were silenced with two or three individual siRNAs, and the average PM density is shown as red line. NT siRNA served as negative and the corrector VX-809 as positive controls (*n* = 3–6). **(B)** Schematic depiction of the assay (upper panel) and representative traces (lower panel) of rΔF508-CFTR function assayed by halide-sensitive YFP quenching in CFBE cells. Knockdown was achieved with two individual siRNAs per indicated gene, and the measurement was performed after 1.5 h chase at 37°C. The ΔF508-CFTR function was measured by determining the YFP quenching kinetics in response to extracellular iodide addition in the presence of forskolin (10 μM), IBMX (250 μM), cpt-cAMP (250 μM), and genistein (50 μM). **(C)** The effect of the indicated gene knockdown on the function of rΔF508-CFTR after 1.5 h chase at 37°C as determined by halide-sensitive YFP quenching (*n* = 3). **(D)** Correlation between the PM density and functional increase of rΔF508-CFTR after knockdown of Yor1-ΔF670 modifier homologs as determined in panels A and C. **(E, F)** The effect of knockdown with two individual siRNAs per gene on PM stability (E, *n* = 4) and functional stability (F, *n* = 3) of rΔF508-CFTR after 1.5 and 3 h chase at 37°C. * *p* < 0.05, ** *p* < 0.01, *** *p* < 0.001. Error bars indicate standard error of the mean (SEM) of 3–6 independent experiments. The underlying data of panels A, C, E, and F can be found in S1 Data.

To test for functional conservation among human homologs of *S*. *cerevisiae* genes found to regulate Yor1-ΔF670 biogenesis, we used the human CF bronchial epithelial CFBE41o- (or CFBE) cell line with *CFTR*^*ΔF508/ΔF508*^ genetic background with no detectable CFTR protein expression as a model [47]. CFBE cells were engineered to express inducible WT- and/or ΔF508-CFTR-3HA as described [17,48]. As an indirect measure of biogenesis and/or peripheral stability of ΔF508-CFTR, first the PM density of the mutant channel was determined by cell surface ELISA in polarized CFBE after siRNA-mediated knockdown of the putative target genes. Two or three nonoverlapping siRNA sequences were used individually for each candidate gene to discriminate possible off-target effects. SiRNA silencing of Yor1-ΔF modifier homologs increased the PM density of the low-temperature (26°C) rescued ΔF508-CFTR (rΔF508-CFTR) by up to ~3-fold, representing ~8% of the WT CFTR PM density, similar to the level achieved by the FDA-approved corrector, VX-809 (**Fig 2A**). A 50% increase in the PM density of rΔF508-CFTR for at least two siRNAs in comparison to nontargeted (NT) siRNA were considered as criteria for further investigation. These were met by siRNA-mediated silencing of *SKIV2L* (*SKI2* in *S*. *cerevisiae*), *TTC37* (*SKI3*), *EXOSC10* (*RRP6*), *RPL12* (*RPL12A*), *POMP* (*UMP1*), and *TBC1D22B* (*GYP1*) (**Fig 2A**).

To determine whether the biochemical rescue of rΔF508-CFTR correlates with a gain-of-function phenotype, the PM conductance of CFBE cells was measured by the halide-sensitive YFP quenching assay in CFBE cells stably expressing the halide-sensitive YFP-H148Q/I152L/F46L, using a fluorescence plate reader [19]. The iodide influx-mediated YFP quenching was determined after maximal activation of the temperature rΔF508-CFTR with cAMP-dependent protein kinase A (PKA) agonists forskolin (Frk), 3-Isobutyl-1-methyl-xanthine (IBMX), and 8-(4-Chlorophenylthio)-adenosine-3',5'-cyclic monophosphate (cpt-cAMP) in combination with the potentiator genistein (gen) (**Fig 2B**). Induction of rΔF508-CFTR expression strongly increased the halide conductance of CFBE epithelia that was further augmented by knockdown of some of the Yor1-ΔF modifier gene homologs (**Figs 2B, 2C, S2A and S2B**). Knockdown of *RPL12* or *SKIV2L*, by two independent siRNAs, increased the mutant channel PM function by >50%. Notable, *RPL12* knockdown induced ~270% transport activity of the NT control (**Fig 2C**).

*RPL12* knockdown, similar to VX-809, proportionally increased the PM density and function of rΔF508-CFTR (**Fig 2D**). In contrast, silencing of *SKIV2L* and most of the other genes tested, evoked a more substantial gain in PM density in comparison to function, suggesting the preferential escape of partially folded, poorly functional ΔF508-CFTR molecules from the ER (**Fig 2D**). The inference that *RPL12* knockdown enhanced the rΔF508-CFTR conformational stability was supported by measuring the PM turnover of rΔF508-CFTR. The rΔF508-CFTR removal from the PM was ~5-fold faster (T_1/2_ ~2 h) in comparison to its WT counterpart (T_1/2_ of >10 h) after exposure to 37°C, which can be attributed to the channel unfolding, reflected by its increased protease susceptibility and ubiquitination that largely accounts for the accelerated internalization, lysosomal targeting, and attenuated recycling [11,49]. *RPL12* knockdown considerably slowed down the rΔF508-CFTR biochemical and functional turnover at the PM (**Fig 2E and Fig 2F**). Jointly, these observations suggest that *RPL12* deficiency promotes rΔF508-CFTR functional PM expression by facilitating the biogenesis and enhancing peripheral stability of the mutant.

Considering that the modifier genes of the Yor1-ΔF/ΔF-CFTR processing defect can contribute to the regulation of translation, RNA processing, and vesicle transport, we tested the possibility that silencing of *TBC1D22B*, *RPL12*, *EXOSC10*, *SKIV2L*, *POMP*, or *TTC37* genes can alter the cellular expression of native and conformationally defective membrane proteins in general. Ablation of these genes, however, did not increase the Ca^2+^-activitated TMEM16A Cl- channel activity, nor the transferrin receptor (TfR) or the conformationally defective, mutant megalencephalic leukoencphalopathy with subcoritical cyst 1 (MLC1-S280L) PM densities, as determined by the halide-sensitive YFP quenching assay [48], transferrin-HRP binding, or cell surface density measurement of MLC1-S280L, respectively (**S2C–S2E Fig**). Thus, silencing of these Yor1-ΔF deletion suppressor proteins does not universally influence PM protein biogenesis.

### SiRNA-Mediated Knockdown of *RPL12* Increases the Expression of Functional ΔF508-CFTR at the Cell Surface

**Fig 3:**
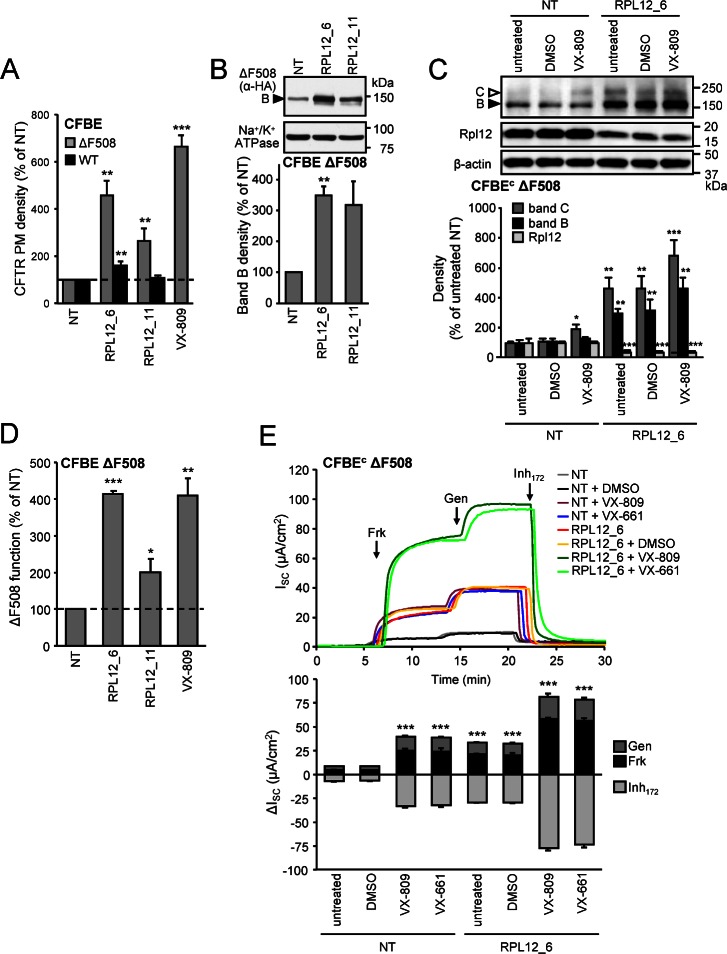
SiRNA-mediated silencing of *RPL12* increases ΔF508-CFTR expression, PM density and function at 37°C. **(A)** PM density of WT and ΔF508-CFTR upon *RPL12* knockdown in CFBE at 37°C (*n* = 3). **(B)** Effect of *RPL12* knockdown on the expression pattern of ΔF508-CFTR determined by immunoblotting in CFBE (upper panel). CFTR was visualized using anti-HA antibody, anti-Na^+^/K^+^-ATPase served as loading control. Densitometric analysis (lower panel, *n* = 3) of the core-glycosylated (band B, filled arrowhead) ΔF508-CFTR is expressed as percent of controls transfected with NT siRNA (*n* = 3). **(C)** The effect of *RPL12* knockdown, VX-809 treatment (3 μM, 24 h) or combination of both on the expression pattern of ΔF508-CFTR expressed constitutively in CFBE cells (CFBE^c^). Immunoblots (upper panel) were probed with antibodies against CFTR (10B6.2), Rpl12, and β-actin as a loading control. Expression levels of Rpl12 and the core- (band B, filled arrowhead) or complex-glycosylated (band C, empty arrowhead) ΔF508-CFTR were quantified by densitometry and are expressed as a percentage compared to controls transfected with NT siRNA (lower panel, n = 3). **(D)** *RPL12* knockdown increases the function of ΔF508-CFTR at physiologic temperature as determined by halide sensitive YFP quenching assay (*n* = 3). **(E)** Representative short-circuit current (I_sc_) recordings (upper panel) and quantification of the changes (ΔI_sc_, *n* = 5, lower panel) in CFBE^c^ monolayers expressing ΔF508-CFTR after siRNA-mediated *RPL12* knockdown or NT siRNA transfection. CFTR mediated currents were induced by sequential acute addition of Frk (10 μM) and gen (50 μM) followed by CFTR inhibition with inhibitor_172_ (10 μM) in the presence of a basolateral-to-apical chloride gradient after basolateral permeabilization with amphotericin B (100 μM). The values in D and E were normalized to account for the increase in ΔF508-CFTR mRNA upon siRPL12_6 treatment. *p < 0.05; **p < 0.01; ***p < 0.001. Error bars show SEM of 3–5 independent experiments. The underlying data of panels A–E can be found in S1 Data.

To examine the effects of *RPL12* silencing at physiological temperature, the ΔF508-CFTR function at the PM was studied in polarized CFBE monolayers. Both siRNAs decreased the *RPL12* protein and mRNA expression by ~50% and ~40%, respectively (**S3A and S3B Fig**). In parallel, the ΔF508-CFTR PM density was increased by 2.5–5-fold relative to the NT siRNA treated cells (**Fig 3A**). The ΔF508-CFTR PM density was comparably augmented in nonpolarized HeLa cells, suggesting that the *RPL12* knockdown effect is not CFBE-specific and independent of CFTR polarized expression (**S3C Fig**). *RPL12* knockdown also enhanced the PM density of WT-CFTR in CFBE and HeLa cells (**Figs 3A and S3C**), probably by increasing the limited maturation efficiency of the WT channel [17,50].

To distinguish whether *RPL12* knockdown promotes the accumulation of the mature, complex-glycosylated (band C) ΔF508-CFTR in post-ER compartments, or causes the channel redistribution from intracellular pools to the PM, the expression level of complex-glycosylated ΔF508-CFTR was determined by immunoblot (IB) analysis. Since we were unable to visualize the complex-glycosylated form in the inducible CFBE expression system (**Fig 3B**), these experiments were also performed in CFBE cells constitutively expressing the channel at higher level, while preserving the hallmarks of ΔF508-CFTR misprocessing (CFBE^C^) [51,52]. In this model, the *RPL12* knockdown increased the abundance of the complex-glycosylated ΔF508-CFTR (**Fig 3C**). Interestingly, *RPL12* knockdown led to the steady-state accumulation of the core-glycosylated ΔF508-CFTR (band B) as well (**Fig 3B and Fig 3C**).

In accord with the increased PM expression, *RPL12* silencing augmented the halide conductance (**Fig 3D**) and the Frk-stimulated short-circuit current (I_sc_) by up to 5-fold (**Fig 3E**). This was further enhanced by addition of the potentiator gen in the constitutive ΔF508-CFTR expressors (**Fig 3E**). In contrast, *RPL12* knockdown had little effect on the function of WT-CFTR in CFBE (**S3D Fig**).

### *RPL12* Knockdown and VX-809 Additively Increase the Functional Expression of ΔF508-CFTR in Immortalized and Primary Human Bronchial Epithelia

**Fig 4:**
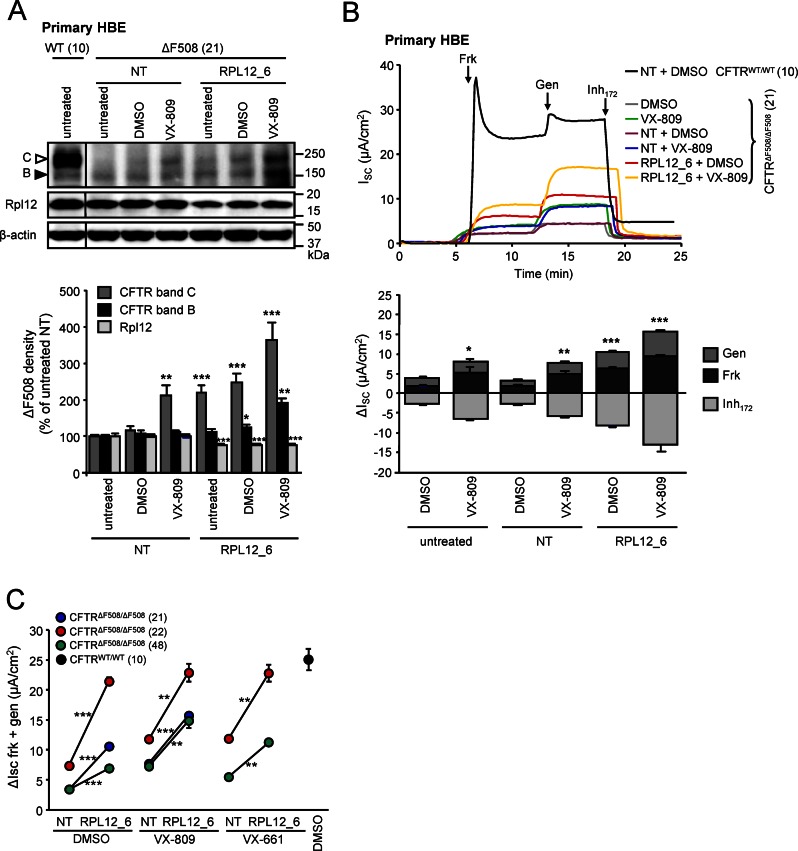
*RPL12* knockdown is additive with VX-809 in primary CF human bronchial epithelia (HBE). **(A)** The effect of *RPL12* knockdown, VX-809 treatment (3 μM, 24 h) or combination of both on the expression pattern of endogenous ΔF508-CFTR in HBE cells isolated from one CF patient with *CFTR*^*ΔF508/ΔF508*^ genotype (upper panel, patient code 21). Immunoblots were probed with antibodies against CFTR (1:1 mixture of UNC antibodies 570 and 596), Rpl12, and β-actin as a loading control. Expression levels of Rpl12 and the core- (band B, filled arrowhead) or complex-glycosylated (band C, open arrowhead) ΔF508-CFTR were quantified by densitometry and are depicted as a percentage compared to controls transfected with NT siRNA (lower panel, *n* = 3). **(B)** Representative I_sc_ recordings (upper panel) and quantification of the changes (ΔI_sc_, lower panel) after siRNA-mediated *RPL12* knockdown, NT siRNA or mock transfection in HBE cells homozygous for ΔF508-CFTR CFTR (patient code 21, *n* = 3). A representative I_sc_ trace of HBE with *CFTR*^*WT/WT*^ genotype is shown for comparison (upper panel). **(C)** Quantification of the Frk- and gen-stimulated current (Δ_Isc_ Frk + gen) in HBE isolated from three different homozygous ΔF508 CF patients (21, 22, and 48) or one WT-CFTR donor (10) transfected with RPL12_6 or NT siRNA for 21 days alone or in combination with VX-809 (3 μM, 24 h) (C) and VX-661 (3 μM, 24 h). CFTR mediated currents were induced by sequential acute addition of Frk (10 μM) and gen (50 μM) followed by CFTR inhibition with inhibitor 172 (Inh_172_, 10 μM) in the presence of a basolateral-to-apical chloride gradient. *p < 0.05; **p < 0.01; ***p < 0.001. Error bars show SEM of three independent experiments. The underlying data of panels A–C can be found in S1 Data.

To confirm the relevance of *RPL12* silencing on the rescue of ΔF508-CFTR misprocessing, we used primary human bronchial epithelia (HBE) isolated from five CF patients with *CFTR*^*ΔF508/ΔF508*^ genotype. CF-HBE were cultured on permeable filter supports at air–liquid interface for 3 wk and transfected with either NT or RPL12_6 siRNA every 7 d, resulting in a decrease of Rpl12 protein expression by ~25% (**Fig 4A**). Alternatively, RPL12 silencing was achieved by a single transfection with a double-stranded Dicer-substrate siRNA [53] (**S4A Fig**). The chronic knockdown of *RPL12* did not alter tight junction formation, the differentiation of globlet and ciliated cells and the development of transepithelial resistance of the monolayers (**S4D Fig and S4E Fig**).

The effect of *RPL12* siRNA alone or in combination with VX-809 was determined on Frk plus gen-activated and inhibitor 172 (Inh_172_)-sensitive I_sc_ of ΔF508-CFTR in CFBE and CF-HBE. VX-809-mediated ΔF508-CFTR correction was additive with *RPL12* knockdown, increasing the maximal I_sc_ to ~50% of WT-CFTR in CFBE (**Figs 3E and S3D**). As expected, a large variation of the I_sc_ was observed in CF-HBE cells from individual patients (**Figs 4B, 4C, S4B and S4C**). *RPL12* silencing enhanced the PKA-activated current by a mean ∼2.2-fold (range 1.1–3.1-fold) in the presence of the potentiator gen. This corresponds to ∼35.7% of the WT-CFTR current, measured in HBE isolated from non-CF lungs (**Figs 4C, S3E and S4C**). VX-809 treatment alone increased the ΔF508-CFTR current by ∼2.1-fold (range 1.6–2.7-fold), representing ∼27.7% of the WT-CFTR current as observed before [54] (**Figs 4C, S3E and S4C**). Combination of *RPL12* knockdown with VX-809 treatment augmented the Frk- and gen-stimulated I_sc_ by ∼4.0 fold (range 3.1–4.9-fold), representing 54.4% of WT-CFTR in HBE cells (**Figs 4C, S3E and S4C**).

*RPL12* knockdown also increased the amount of complex-glycosylated ΔF508-CFTR in HBE, similar to that in CFBE (**Fig 4A**). Treatment with VX-809 alone led to ∼2-fold enhanced expression of the complex-glycosylated form in both CFBE and CF-HBE cells. The rescue effect of VX-809 was at least doubled upon *RPL12* silencing, indicated by ~6- and ~4-fold increase of the band C abundance of ΔF508-CFTR in CFBE and HBE, respectively (**Figs 3C and 4A**). These findings support the notion that distinct mechanisms are responsible for the *RPL12* knockdown-mediated rescue and VX-809-dependent correction of the ΔF508-CFTR functional expression defect in both CFBE and HBE cells.

### *RPL12* Knockdown Increases the ΔF508-CFTR Folding Efficiency at the ER

**Fig 5:**
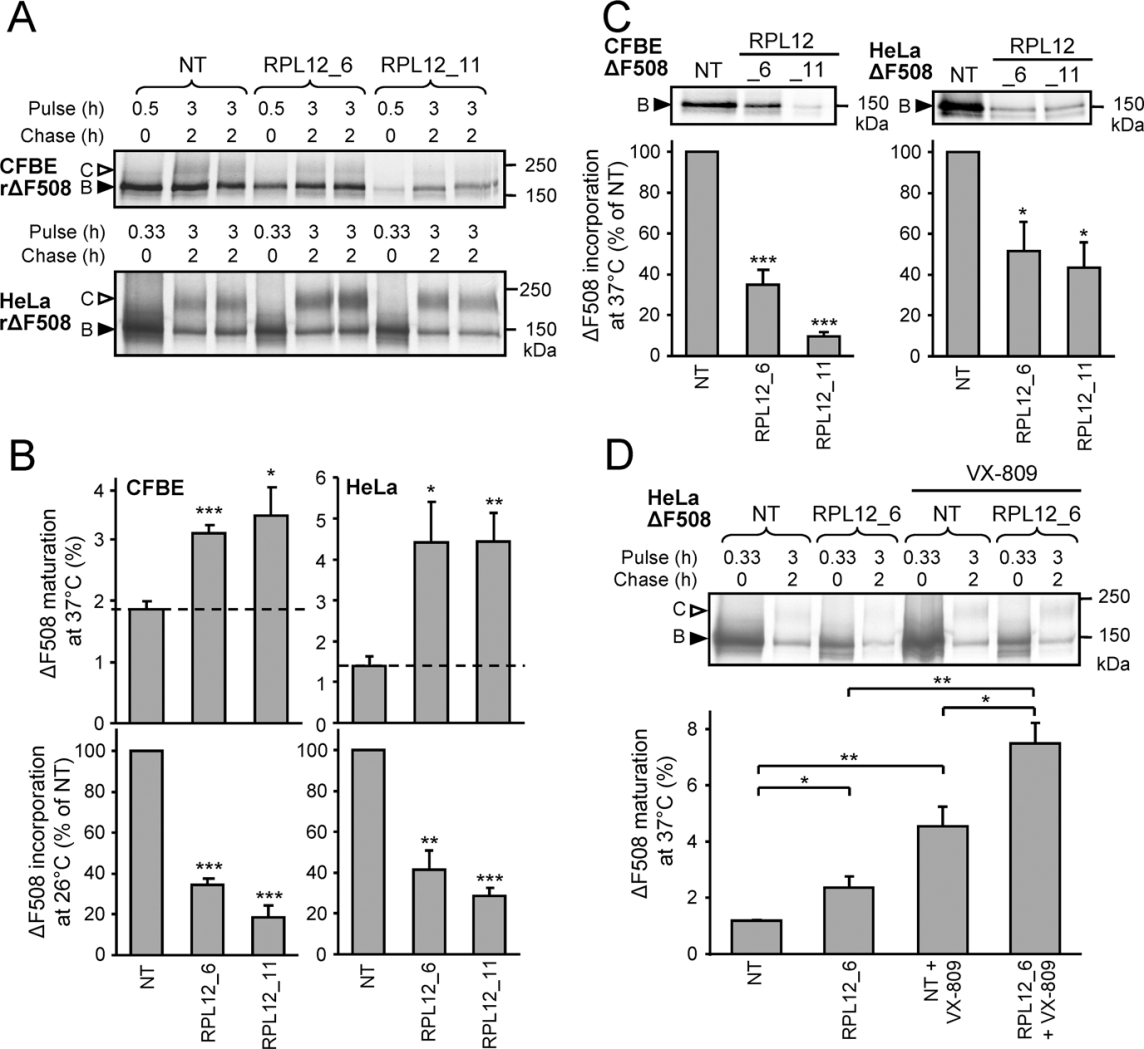
*RPL12* knockdown increases the conformational maturation of ΔF508-CFTR at the ER. **(A)** Determination of ΔF508-CFTR translation and ER folding efficiency by metabolic pulse chase in CFBE and HeLa cells transfected with *RPL12* or NT siRNA. Labeling with [^35^S]-methionine and [^35^S]-cysteine was performed to measure translation (30 min in CFBE or 20 min in HeLa at 26°C) or maturation efficiency (for 3 h at 26°C followed by 2 h chase at 37°C) in duplicates. **(B)** The folding efficiency of ΔF508-CFTR after 2 h chase at 37°C in CFBE (left upper panel) and HeLa cells (right upper panel) was determined by calculating the percent of pulse-labeled immature, core-glycosylated ΔF508-CFTR (B-band, filled arrowhead in A) conversion into the mature, complex-glycosylated form (C-band, empty arrowhead in A) (*n* = 4–6). The total labeling for 3 h was extrapolated from values obtained for 20 or 30 min pulse labeled samples. Quantitative analysis of ^35^S-methionine and ^35^S-cysteine incorporation during the 20 or 30 min labeling period at 26°C into the newly formed ΔF508-CFTR in CFBE (left lower panel) and HeLa cells (right lower panel) (*n* = 3). **(C)** [^35^S]-methionine and [^35^S]-cysteine incorporation during the labeling period at 37°C into the newly formed ΔF508-CFTR in CFBE (left panel, 30 min) and HeLa cells (right panel, 20 min) (*n* = 3). **(D)** Determination of ΔF508-CFTR translation and ER folding efficiency by metabolic pulse chase in HeLa cells transfected with *RPL12* or NT siRNA with or without VX-809. Labeling was performed for 20 min with no chase (0 h chase) or for 3 h followed by 2 h chase, both at 37°C. The folding efficiency of ΔF508-CFTR after 2 h chase at 37°C is depicted in the lower panel (*n* = 3) and was calculated based on the extrapolated total labeling for 3 h from values obtained for the 20 min pulse labeled samples without chase. Radioactivity was quantified based on phosphoimage analyses and not by densitometry of the autoradiographs used for illustration. *p < 0.05, **p < 0.01, ***p < 0.001. Error bars show SEM of 3–6 independent experiments. The underlying data of panels B–D can be found in S1 Data.

We postulated that *RPL12* knockdown may enhance the conformational maturation efficiency of non-native ΔF508-CFTR at the ER and/or stabilize the native-like conformation of the mutant, delaying its cell surface removal. The low folding efficiency of the nascent ΔF508-CFTR chains were measured by the conversion efficiency of the core-glycosylated ΔF508-CFTR into complex-glycosylated form during an extended radioactive pulse labeling (3 h) and chase (2 h) at 37°C. To measure the total [^35^S]-methionine and [^35^S]-cysteine incoporation during the pulse, while minimizing the degradation of core-glycosylated forms, the pulse duration was reduced to 30 and 20 min in parallel samples of CFBE and HeLa cells, respectively, (**Fig 5A**). *RPL12* knockdown increased the ΔF508-CFTR ER folding efficiency from ~1.9% to ~3.2% in CFBE and from ~1.4 to ~4.4% in HeLa cells (**Fig 5A and Fig 5B**), while it decreased the radioactive labeling of the core-glycosylated ΔF508-CFTR by 50–80% both at 26°C and 37°C (**Fig 5A–5C**) without reducing the CFTR transcript level (**S5A Fig**). We obtained similar results by using a shorter pulse-labeling time (10 min) (**S5C Fig**). Combination of *RPL12* knockdown with VX-809 additively increased the ΔF508-CFTR maturation efficiency from 4.5% to ~7.5% relative to that in the presence of VX-809 alone, at 37°C (**Fig 5D**), consistent with nonoverlapping mechanisms of action.

### *RPL12* Silencing Cannot Indiscriminately Rescue the Processing Defect of PM Proteins

Selectivity of the *RPL12* knockdown effect was determined by measuring its impact on the cell surface density and turnover of a panel of PM proteins as surrogate readouts of their conformational stability [11,50,55,56]. We chose mutations in the vasopressin 2 receptor (V2R-Y128S) [56,57], MLC1-P92S and -S280L [58], and the human Ether-à-go-go-Related Gene (hERG-G601S) [55], which cause diabetes insipidus (V2R), megalencephalic leukoencephalopathy (MLC1), and long QT type 2 syndrome (hERG), respectively, due to conformational defects, misprocessing and accelerated PM turnover of the respective membrane proteins [55–60].

**Fig 6:**
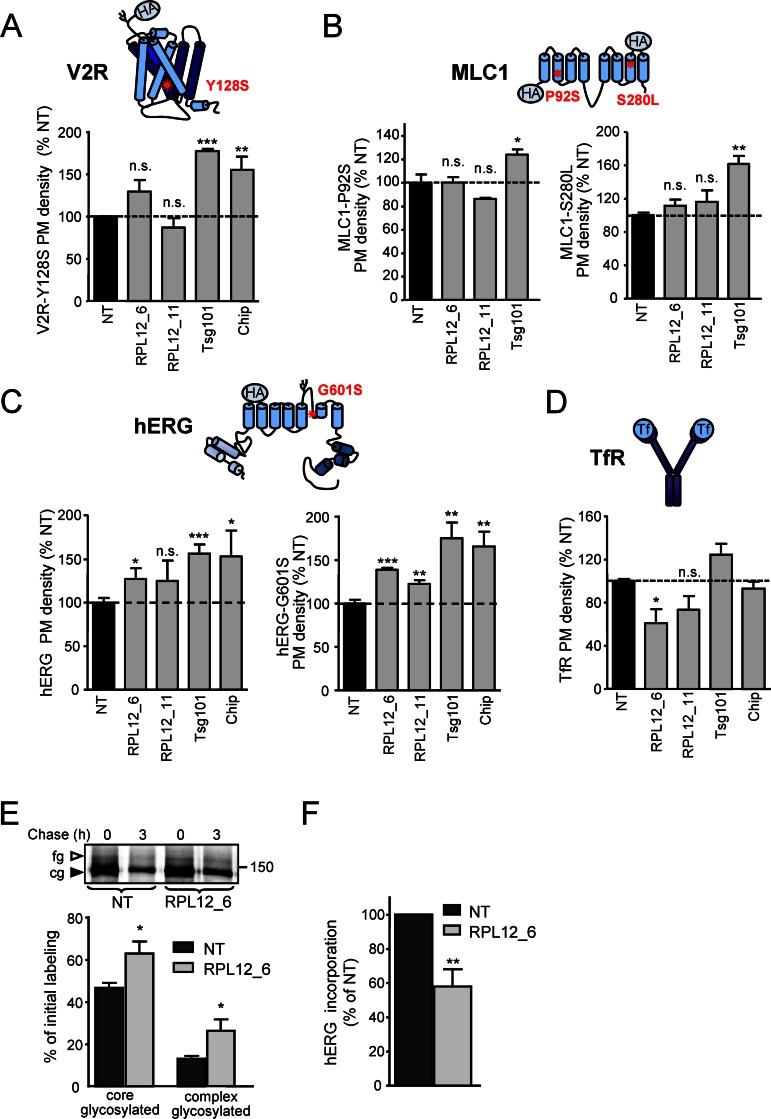
*RPL12* knockdown increases the maturation efficiency of some misfolded membrane proteins. **(A–D)** PM densities of the indicated proteins were measured by cell surface ELISA in NT or *RPL12* siRNA transfected HeLa cells. In A–C, cells stably expressing extracellular HA-epitope tagged V2R-Y128S (A, *n* = 3), MLC1-P92S and S280L (B, *n* = 3) or hERG-WT and G601S (C, *n* = 4) were used. The endogenous TfR was labeled with biotin-Tf and detected with neutravidin-HRP (D, *n* = 3). SiRNAs for CHIP and Tsg101 served as positive controls that attenuated degradation of misfolded PM proteins from post-Golgi compartments. **(E)** The maturation efficiency of hERG-G601S was determined by metabolic pulse chase experiment using ^35^S-methinonine and ^35^S-cysteine in HeLa cells transfected with *RPL12* or NT siRNAs. Pulse labeling was performed for 20 min at 26°C, followed by 3 h chase at 37°C. The maturation efficiency of the channel was calculated based on the percent conversion of the pulse labeled core-glycosylated form (cg-form, filled arrowhead) into the complex-glycosylated form (fg, empty arrow head) of hERG-G601S after 3 h of chase (*n* = 4). **(F)** ^35^S-methinonine and ^35^S-cysteine incorporation into newly synthesized hERG-G601S during the 20 min pulse labeling at 26°C (*n* = 4). *p < 0.05; **p < 0.01; ***p < 0.001. Error bars show SEM of 3–4 independent experiments. The underlying data of panels A–F can be found in S1 Data.

Silencing of *RPL12* had no significant effect on PM density and stability of the V2R-Y128S and MLC-P92S or -S280L in HeLa cells (**Figs 6A, 6B, S6A and S6B**). In contrast, knockdown of *RPL12* increased the WT-hERG (~125% of NT) and hERG-G601S (140% of NT) PM densities (**Fig 6C**), without stabilizing the channels at the PM (**S6C Fig**). The effect on steady-state PM density of WT and mutant hERG may be due to their inefficient conformational maturation, estimated to be ~40% and ~15% determined by metabolic pulse chase experiments [61] (**Fig 6E**). *RPL12* knockdown increased the hERG-G601S maturation efficiency from ~15% to ~25% (**Fig 6E**).

Notably, incorporation of radioactive amino acids into newly synthesised hERG-G601S was decreased in *RPL12* knockdown cells (**Fig 6F**). This could be attributed to translational inhibition rather than cotranslational degradation, since the amount of core-glycosylated hERG was increased after a 3 h chase at 37°C (**Fig 6E**). The PM density of the TfR, a type I transmembrane protein [56], was only modestly decreased by *RPL12* knockdown (**Fig 6D**). These results support the hypothesis that the *RPL12* knockdown can preferentially influences the folding efficiency of metastable, multidomain membrane proteins.

### *RPL12* Silencing Enables the Biogenesis of Native-Like ΔF508-CFTR

Compelling evidence indicates that synonymous mutations can influence the folding of polypeptide via defining the available time frame for the cotranslational folding and unfolding during translation in vivo and in vitro [62–65]. If translational elongation slow down can shift the folding equilibrium of newly synthesized multidomain ΔF508-CFTR towards the WT-CFTR conformer by kinetic or thermodynamic means, it is predicted that the thermostability of complex-glycosylated mutant would be increased as compared to the temperature rΔF508-CFTR.

**Fig 7:**
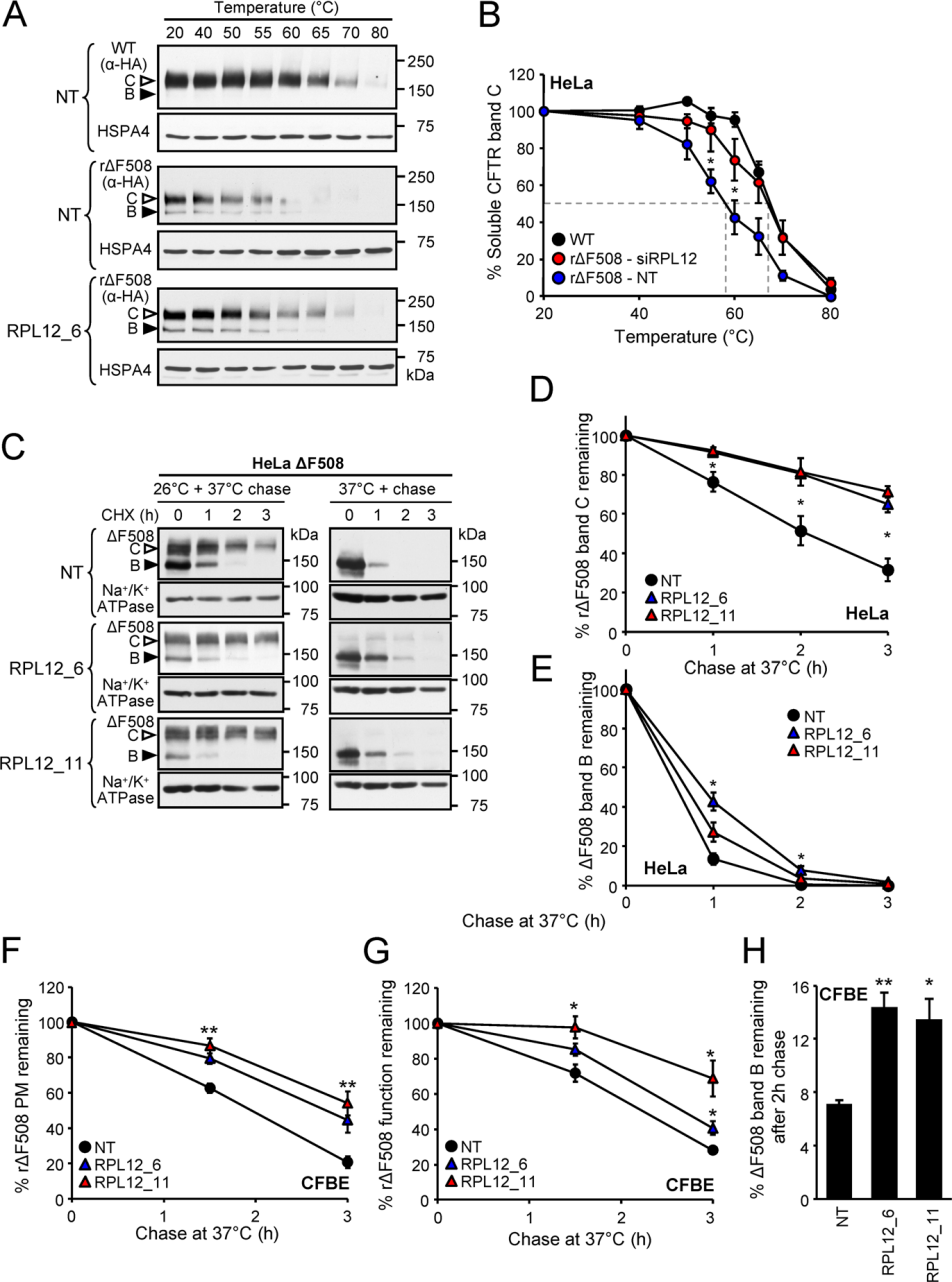
*RPL12* knockdown increases the stability of the core- and complex-glycosylated forms of ΔF508-CFTR. **(A, B)** *RPL12* ablation increases the conformational stability of the solubilised rΔF508-CFTR. The thermoaggregation propensity of rΔF508-CFTR as a surrogate indicator of the channel conformational stability was determined in cell lysates of HeLa cells transfected with *RPL12* or NT siRNA in comparison to WT-CFTR. To minimize the amount of core-glycosylated form (filled arrowhead), cells were treated with CHX (2 h, 100 μg/ml) prior to lysis. Cell lysates were incubated for 15 min at 20°C–80°C followed by the sedimentation of aggregates and visualizing the remaining soluble channels by immunoblotting (A). The complex-glycosylated channel (empty arrowhead) was quantified by densitometry (B, *n* = 3–5). **(C)** Stability of ΔF508-CFTR after (left panel) or without (right panel) low-temperature rescue in HeLa cells upon *RPL12* knockdown was determined by immunoblot with CHX chase. **(D, E)** The complex-glycosylated (D, open arrowhead in C) or core-glycosylated CFTR (E, filled arrowhead in C) disappearance was quantified by densitometry and is expressed as percent of the initial amount (*n* = 3). **(F, G)** The effect of *RPL12* silencing on the PM stability (F, *n* = 4) and functional stability (G, n = 3) of rΔF508-CFTR after 1.5 and 3 h chase at 37°C. Same values as in Fig 2E and 2F depicted as chase time-dependent percent remaining. **(G)** The effect of *RPL12* knockdown on the stability of metabolically labeled core-glycosylated ΔF508-CFTR in CFBE. Labeling was performed for 3 h at 26°C followed by chase for 2 h at 37°C. *p < 0.05; **p < 0.01; ***p < 0.001. Error bars show SEM of 3–5 independent experiments. The underlying data of panels B and D–H can be found in S1 Data.

The conformational stability of CFTR channels was measured by determining the thermal denaturation temperature, which induced 50% (T_50%_) conversion of detergent solubilised CFTR into SDS-resistant aggregates [66]. Cell lysates, obtained from rΔF508 or WT CFTR CFBE expressors were heat-denatured at 20°C–80°C and the aggregation resistant, complex-glycosylated CFTR pool was quantified by immunoblotting. *RPL12* knockdown increased the T_50%_ of the complex-glycosylated rΔF508-CFTR from ~58°C to 67°C, a value that is close to the T_50%_ of WT-CFTR (**Fig 7A and Fig 7B**). This observation suggests that *RPL12* silencing promotes the accumulation of conformationally more stable complex-glycosylated rΔF508-CFTR, conceivably by shifting the mutant folding energetic towards the WT, an inference substantiated by increased metabolic stability of the rΔF508-CFTR, determined by CHX chase and immunoblotting in HeLa cells (**Fig 7C and Fig 7D**). *RPL12* silencing also delayed the rΔF508-CFTR biochemical and functional turnover at the PM in CFBE, as determined by cell surface ELISA and halide-sensitive YFP assays (**Fig 7F and Fig 7G**).

Surprisingly, *RPL12* knockdown delayed the turnover of the immature ΔF508-CFTR. The core-glycosylated form T_1/2_ was decreased from ~30 to ~50 min in HeLa and CFBE measured by CHX chase and immunoblotting. (**Figs 7C, 7E and S5D**). Comparable results were obtained by the metabolic pulse-chase technique. After 2 h chase ~14% of the initially labeled immature ΔF508-CFTR remained in the *RPL12* knockdown CFBE cells as compared to ~7% in the NT control at 37°C (**Fig 7H**). Thus, impeded degradation of the core-glycosylated form may contribute to the enhanced ER maturation of ΔF508-CFTR in CFBE cells (see also **Fig 3B and 3C**).

Collectively, these observations suggest that the increased folding propensity of ΔF508-CFTR in the presence of *RPL12* down-regulation account for the increased PM density, function, biochemical, and functional stability, as well as reduced potentiator-dependent fractional activation of the I_sc_ carried by ΔF508-CFTR (**S5C Fig and S5D Fig**).

### Slow-Down of Translational Elongation Rate by Multiple Means Partially Reverts the Folding and Processing Defects of ΔF508-CFTR

**Fig 8:**
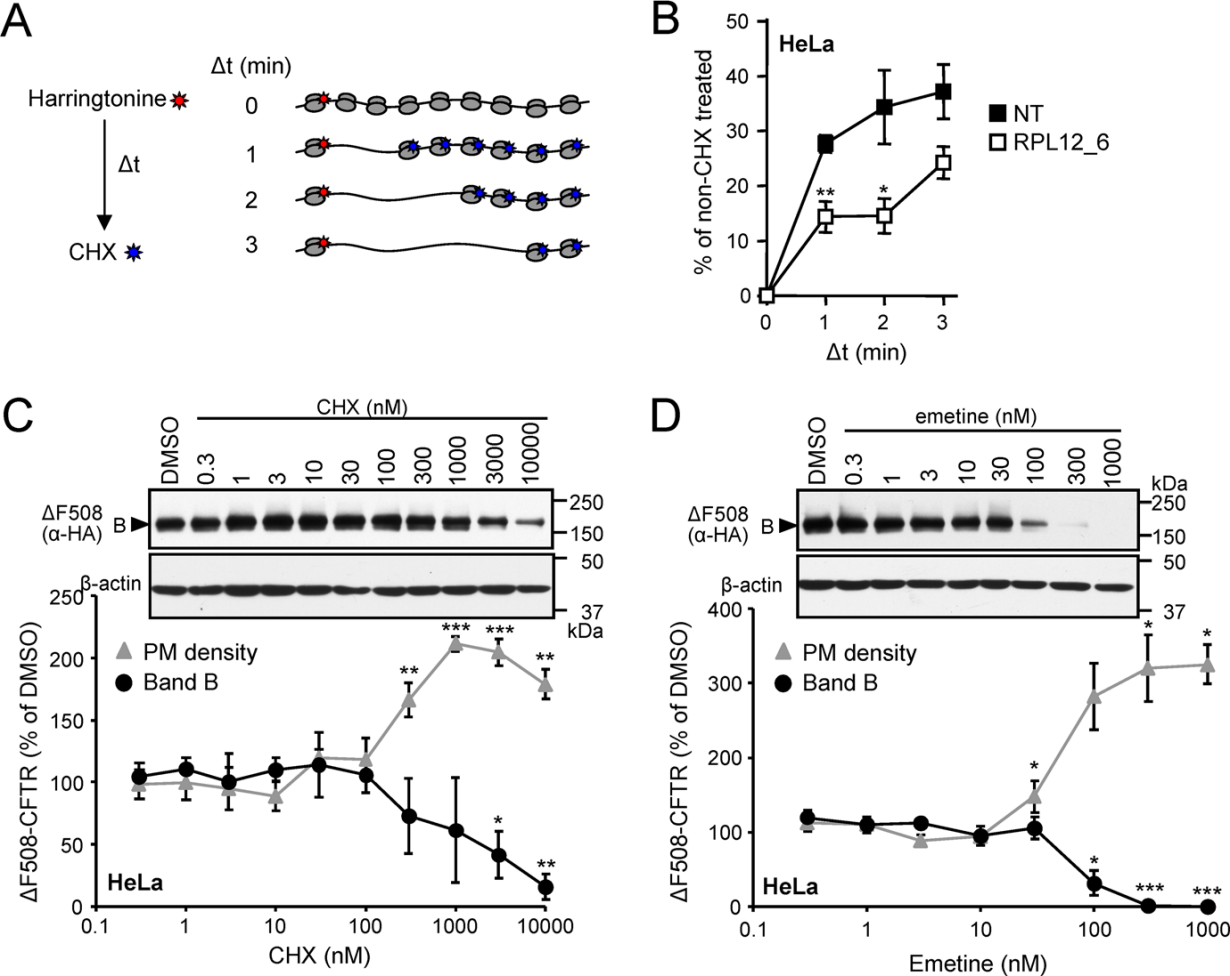
Slow-down of translational elongation rate partially corrects ΔF508-CFTR folding defects. **(A, B)** Schematic depiction of the run-off elongation experiment (A) and relative translation elongation rates in HeLa cells transfected with *RPL12* or NT siRNA (B). The values were normalized to cells in which the initiation was stopped with harringtonine (2 μg/ml), but the run-off elongation was allowed to proceed without CHX inhibition (*n* = 4). **(C, D)** PM density and total expression of ΔF508-CFTR after 24 h treatment with increasing concentrations of CHX (C) or emetine (D) in HeLa. ΔF508-CFTR expression was visualized by immunoblot using anti-HA antibody, anti-β-actin antibody served as loading control (upper panels). Densitometric analysis of core-glycosylated ΔF508-CFTR expression and PM density are expressed as a percentage of DMSO controls (lower panels, *n* = 3). *p < 0.05; **p < 0.01; ***p < 0.001. Error bars show SEM of 3–4 independent experiments. The underlying data of panels B–D can be found in S1 Data.

RPL12 knockdown causes translational elongation slow down in yeast [67,68]. Consistently, the reduced pulse labeling of ΔF508-CFTR and HERG-G601S suggested that *RPL12* silencing may also impede the translational elongation rate in eukaryotes (**Figs 5B, 5C, 6F and S5C**). Due to insufficient radioactive signal incorporation into the ΔF508-CFTR nascent chain in CFBE cells, we monitored the global kinetics of run-off elongation [69] in HeLa cells after *RPL12* silencing. To this end, translation initiation was inhibited with harringtonine [70]. Then run-off elongation was measured in the presence of [^35^S]-methinonine and [^35^S]-cysteine and terminated with cycloheximide (CHX) [71] after 1–3 min (**Fig 8A)**. Quantification of radioactive nascent chains synthesis by phosphorimage analysis showed a considerable reduction in the global translational elongation rate in *RPL12* knockdown cells (**Fig 8B**). Importantly, slowing down global translational elongation in Fisher rat thyroid (FRT) cells by low concentration of CHX also induced the accumulation of the complex-glycosylated ΔF508-CFTR and partial restoration of the PM chloride conductance [72].

To confirm that pharmacological inhibition of translational elongation can partially rescue ΔF508-CFTR processing in CFBE cells, we used low-concentrations CHX or emetine. Both translational elongation inhibitors elicited a dose-dependent increase in the PM density of ΔF508-CFTR, which correlated with the decreased expression of the core-glycosylated form as a consequence of partial translational inhibition (**Fig 8C and Fig 8D**). These results confirmed and extended previous observations [72], suggesting that the channel folding is favoured at a slower translational elongation rate regardless of whether it is achieved by pharmacological [72] or genetic means as shown by RPL12 ablation (**Fig 8B**).

### SiRNA-Mediated Silencing of Ribosomal Stalk Proteins or the Elongation Factor 2 Increases the Functional Expression and Stability of ΔF508-CFTR

**Fig 9:**
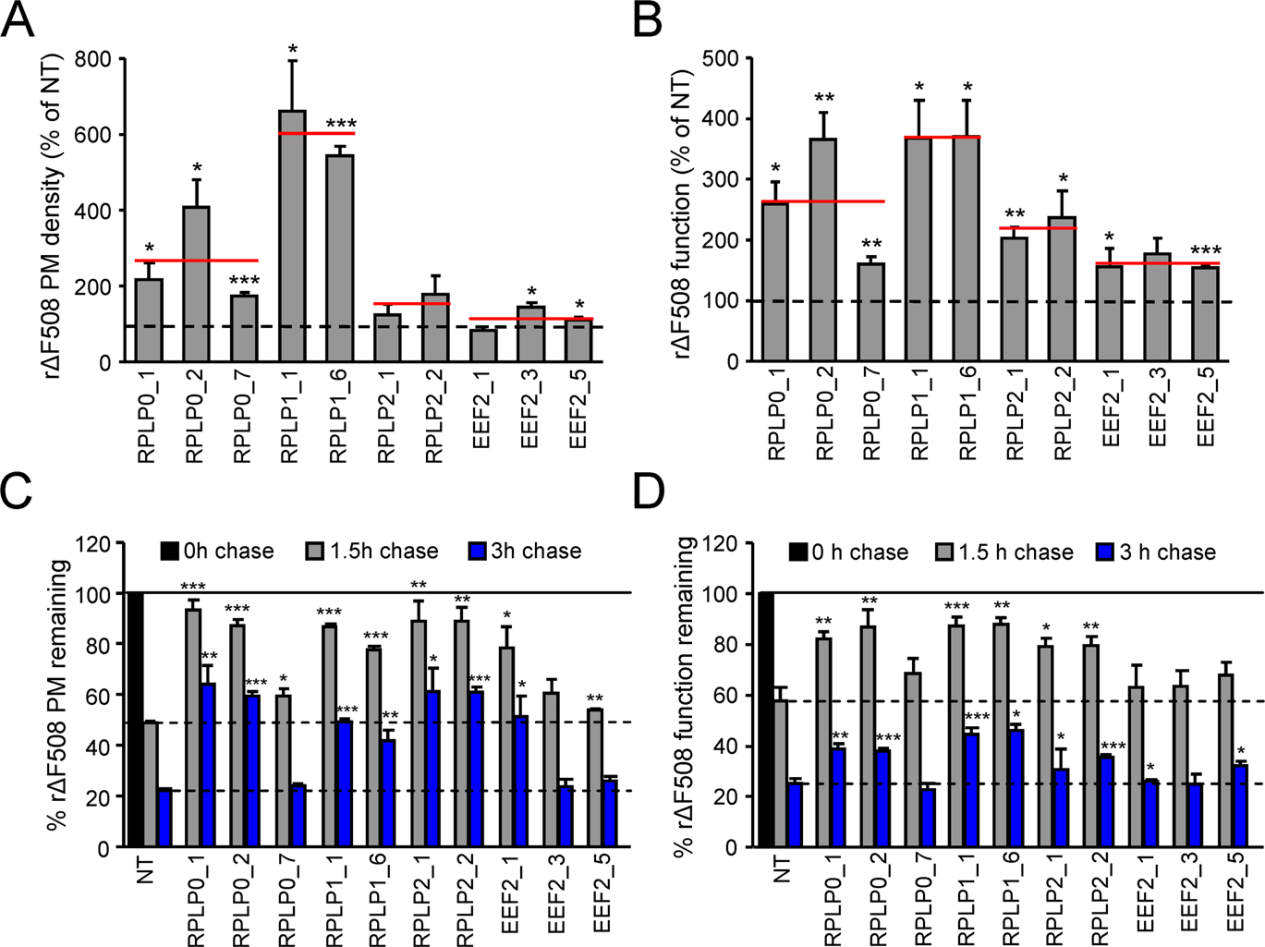
SiRNA-mediated silencing of ribosomal stalk proteins enhances the PM density, function, and stability of ΔF508-CFTR. **(A, B)** The effect of ribosomal stalk protein (RPLP0—P0, RPLP1—P1, and PRPL2—P2) and eEF-2 knockdown on the PM density (A) and function (B) of rΔF508-CFTR in CFBE. The indicated ribosomal proteins were silenced with two to three individual siRNAs and the mean PM density or function is shown as a red line. The values are expressed as percent of NT siRNA controls (*n* = 3). **(C, D)** The effect of ribosomal stalk protein and eEF-2 knockdown on the PM (C, *n* = 3) and functional stability (D, *n* = 3) of rΔF508-CFTR after 1.5 and 3 h chase at 37°C.*p < 0.05, **p < 0.01, ***p < 0.001. Error bars show SEM of three independent experiments. The underlying data of panels A–D can be found in S1 Data.

Deletion of the *RPL12A* and *RPL12B* genes in yeast leads to a defective assembly of the ribosomal stalk, disrupting the normal stoichiometry of P1 and P2 isoforms, which along with P0, and the 26S (28S in humans) rRNA, interact with Rpl12 to form the GTPase-associated center (GAC) [67,73,74]. The stalk has been shown to participate in the translocation mechanism and to bind the eukaryotic elongation factor 2 (eEF-2), leading to the hypothesis that P-proteins act as GTPase-activators in conjunction with eEF-2 to increase the translational elongation rate [74–77]. To support the notion that compromised integrity of the ribosomal stalk could increase the functional expression of ΔF508-CFTR, the expression levels of P0 (RPLP0), P1 (RPLP1), P2 (RPLP2), and eEF-2 proteins were reduced by siRNA-mediated knockdown in CFBE. Two or three siRNA sequences were used for each gene, reducing the mRNA expression by ~10%–60% after 5 d in polarized CFBE (**S7A–S7D Fig**). Transfection of these siRNAs increased the PM density and function of rΔF508-CFTR by up to 5-fold as compared to NT siRNA and indicated a good correlation between the knockdown efficiencies and the gain of PM density and function of the mutant (**Figs 9A, 9B, S7F and S7G**). Importantly, P0, P1, P2, and eEF2 knockdown conformationally stabilized the mutant, as indicated by the slower biochemical and functional PM turnover of the rΔF508-CFTR (**Fig 9C and Fig 9D**).

Considering that reduced translational elongation rate following RPL12 knockdown is associated with the reduction of the ER load, and this may influence the protein homeostasis (proteostasis) network activity [23], we measured the expression level of molecular chaperones and cochaperones, critical determinants of the proteostasis network folding capacity [11]. The expression level of HSP90A, HSPA8, AHA1, STIP1, DNAJA1, and BAG1 however, remained unaltered upon *RPL12* ablation (**S8 Fig**), suggesting that the ratio between the total pool of synthesized proteins and these chaperones and cochaperones was not affected by *RPL12* knockdown.

As an alternative method to test the influence of the ER load on cotranslational folding of ΔF508-CFTR, we partially inhibited translation initiation by reducing one of the core translation initiation factors, the cap-binding protein eIF4E, expression using siRNA-mediated knockdown. Reduction of the eIF4E mRNA by ~50% (**S7E Fig**) decreased the pulse-labeled pool of ΔF508-CFTR by ~30% **(S7L Fig)**. This led to a 20%–30% increase in the rΔF508-CFTR PM density and function without altering its fast PM turnover or functional stability (**S7H–S7K Fig**). These results are at variance with observation obtained in FRT cells upon inhibiting initiation with hippuristanol, which increased the ΔF508-CFTR activity by ~2.5-fold at the PM [72]. Thus, significant reduction of protein synthesis, including ΔF508-CFTR and the ER load by impeding translational initiation without translational elongation has a modest contribution to the conformational rescue of ΔF508-CFTR, as compared to that of RPL12 silencing in CFBE and failed to provoke the accumulation of native-like, conformationally stabilized rΔF508-CFTR at the PM.

## Discussion

While technology developments significantly improved the identification of the interactome of conformationally defective polypeptides, including the ΔF508-CFTR [28–30], establishing the functional significance of interacting proteins remains a daunting task. One possible approach is offered by global, quantitative analysis of functional gene interaction in yeast models. While it is appreciated that the molecular processes involved in the translation, folding, maturation, and trafficking of integral membrane proteins are evolutionarily conserved [78], the extent to which global, quantitative analysis of gene interaction in a yeast model yields correct predictions of human genetic modifier that enhance the functional rescue of disease-causing target proteins, remains poorly understood [79,80].

Here, we show that gene interaction studies using the yeast ATP-binding cassette family C (ABCC) protein Yor1 incorporating the ΔF508-CFTR homologous mutation ΔF670 can predict novel modifier genes of ΔF508-CFTR biogenesis in respiratory epithelia. Silencing six of the seven genes selected to probe various steps during Yor1-ΔF670 biogenesis that acted as deletion suppressors to augment Yor1-ΔF670-dependent oligomycin resistance [39], also increased PM expression of ΔF508-CFTR in the human CFBE cells. Knockdown of *RPL12*, a component of the large ribosomal subunit stalk, increased both the ER folding efficiency as well as the metabolic and functional stability of the mature, complex-glycosylated ΔF508-CFTR in post-ER compartments and at the PM, respectively. These results jointly indicate the conservation/concordance of gene interaction modules influencing the early biogenesis of Yor1-ΔF670 and ΔF508-CFTR, including mRNA turnover, rRNA processing, and ribosome structural assembly.

In contrast to mammalian cells, the Rpl12 protein in *S*. *cerevisiae* is encoded by a duplicated gene, *RPL12A* and *RPL12B*, which are remnants of a genome duplication event [81]. In the case of *RPL12* and Yor1-ΔF biogenesis, both *RPL12A* and *RPL12B* acted as deletion suppressors, but *RPL12A* knockout yielded a quantitatively greater effect. Deletion of both genes is associated with a severe growth, but not a viability defect and results in a profound decrease in the translational elongation rate, while deletion of either single gene slightly retarded cell growth and elongation [67,68].

Knockdown of Rpl12 protein expression by ~50% considerably decreased the global translational elongation rate as determined by the kinetics of run-off elongation in human CFBE cells (**Fig 8B**). The concomitant increase of ΔF508-CFTR PM density and function is reminiscent of a recent report by Meriin et al., demonstrating that pharmacological attenuation of translational initiation or elongation rate by hippuristanol or CHX, respectively, can partially rescue the PM functional expression defect of ΔF508-CFTR in FRT cells [72]. While the cellular and molecular mechanism of ΔF508-CFTR rescue is not known in FRT cells, here we show that RPL12 silencing increases the ER folding efficiency of the nascent chain and the stability of both the core- and complex-glycosylated forms, as well as the PM resident ΔF508-CFTR channel. The enhanced ER folding efficiency can be attributed, at least in part, to favourably changes in the folding energetics of the mutant, as reflected by the increased thermostability of the final fold, represented by the complex-glycosylated ΔF508-CFTR in *RPL12* ablated cells (**Fig 7B**).

While we do not have a definitive explanation for the significantly increased co- and post-translational folding efficiency and stabilization of ΔF508-CFTR upon translational elongation slow down, similar phenomena have been demonstrated for various polypeptides in silico, in vitro as well as in vivo [62–65,82,83]. Translational rate slow down may help to reduce the amount of misfolded domain intermediates that are resistant to conformational rescue by the proteostasis machinery, influence the stability of folding intermediates of the nascent chain and/or the folding trajectory of ΔF508-CFTR by altering the binding to profolding and/or prodegradative constituents of the ribosome associated quality control machinery [84–87]. The longer residence time of the nascent chain on the ribosome may also shield against degradation and facilitate folding.

The hypothesis that reduced translation elongation increases ΔF508-CFTR folding is consistent with earlier observations showing that slowing the global translational elongation rate by various interventions can improve the folding efficiency of specific proteins in eukaryotic cells. Synonymous codon changes that result in ribosomal pausing can lead to alternate folding pathways, distinct conformations, and can facilitate cotranslational targeting of membrane proteins to the translocon [63,65,88], as has been shown for the multidrug resistance 1 gene product P-glycoprotein [62,64]. In contrast, accelerating translation rate by synonymous codon substitutions in the α-subdomain of WT-CFTR NBD1 resulted in aggregation of the full-length channel [89]. Codon optimization also resulted in conformational changes and impaired function of *Neurospora*, a clock protein [90].

The possibility that global translational attenuation by *RPL12* knockdown can exert a general profolding effect is unlikely but cannot be ruled out. *RPL12* silencing did not shift the expression levels of known CFTR modifier genes such as HSP90A, HSPA8, AHA1, STIP1, DNAJA1, and BAG1, which are molecular chaperones and cochaperones that are known modulators of ΔF508-CFTR folding and PM expression [11]. Furthermore, whereas *RPL12* siRNA increased the PM expression of hERG-G601S and ΔF508-CFTR, and to some extent their WT-counterparts, *RPL12* ablation had no effect on the PM density of V2R-Y128S, as well as MLC1-P92S or -S280L, despite the documented recognition of these misfolded membrane proteins by the ER and PM protein quality control systems [11,55,56,58,91]. Thus, intrinsically slow and inefficient folding and domain assembly of the WT CFTR and hERG, which is further compromised by missense mutations, may sensitize these complex multidomain proteins to rescue upon translational elongation slow down.

Rpl12 is localized in the 60S subunit GAC and interacts with the GTP-bound translation factors [92–94]. Rpl12 binds to the 28S rRNA together with ribosomal protein P0 to constitute the base of the lateral ribosomal stalk (P-stalk), which serves as a binding platform for ribosomal proteins P1 and P2 [67,73,74,95,96]. The stalk is present in all eukaryotic ribosomes and plays an integral role in translation elongation, due to specific interactions of P1 and P2 with eukaryotic elongation factor 1α (eEF-1α) and Rpl12 with eEF-2 [92–94]. Based on evidence in yeast showing disruption of stalk components reduces translation without changing translational fidelity [68,75,76,97], we hypothesize that similar to the knockdown of *RPL12*, silencing of other ribosomal stalk proteins, which inhibits elongation factors recruitment and slows down the translational elongation, allows the development of a native-like ΔF508-CFTR conformation. Consistent with this hypothesis, silencing of P1, P2, P0, or eEF-2 increased the ΔF508-CFTR functional expression and PM stability (**Fig 9**).

In contrast, silencing of one of the core translation initiation factors, the cap-binding protein eIF4E, decreased ΔF508-CFTR synthesis but only slightly increased ΔF508-CFTR PM density without stabilizing the rescued mutant channel biochemically or functionally at the PM. Studies in FRT cells, however, showed that partial inhibition of translation initiation with hippuristanol leads to a substantial increased ΔF508-CFTR function [72]. Thus, the concomitant reduction of the overall ER protein load might be a contributing factor to the increased PM expression and function of ΔF508-CFTR, but is likely insufficient to explain enhanced ER folding efficiency of the mutant channel.

Our results showing that knockdown of different ribosomal stalk proteins increases the folding propensity and function of ΔF508-CFTR provide new insight into targeted slow-down of translational elongation as a possible therapeutic strategy for treating a conformational disease resulting from mutations in an evolutionarily conserved amino acid residue. Whether other ribosomal proteins and ribosome-bound soluble factors are involved in this mechanism or small molecule inhibitors can be identified to mimic the genetic effect of *RPL12* knockdown remain to be established in future work.

From a translational perspective, our findings show that *RPL12* knockdown is additive with the small-molecule corrector VX-809 in primary HBE cells with *CFTR*^*ΔF508/ΔF508*^ genotype. Moreover, the treatment combination restores ΔF508-CFTR function to ~50% of the WT level, a value that is deemed sufficient to alleviate CF clinical manifestations, since heterozygous carriers do not show disease symptoms [98]. Knockdown of other ribosomal stalk proteins and the translational elongation factor eEF-2, similarly result in improved ΔF508-CFTR biogenesis. Thus, ribosomal stalk perturbation represents a potential target for rescuing the ΔF508-CFTR biogenesis in combination with VX-809, which represents one the most efficacious strategies for the correction of ΔF508-CFTR thus far [20]. Future work will clarify the mechanism by which the reduced translational elongation rate via reduced function of the ribosomal stalk could be used to treat ~90% of CF patients afflicted with this most common allele. Furthermore, application of yeast phenomics as part of an integrative research approach should be considered for its potential to discover genotype-phenotype networks that can guide discovery of new therapeutics for patients with CF and possibly other diseases that can be modeled in *S*. *cerevisiae*.

## Materials and Methods

### Ethics Statement

Human lung tissues were obtained from *CFTR*^*WT/WT*^ non-CF and *CFTR*^*ΔF508/ΔF508*^ CF individuals under the protocol and consent form approved by the Institutional Review Board at University of Alabama Birmingham (IRB #X080625002). All adult participants provided informed consent, or a parent or guardian of any child participant provided informed permission on their behalf. All consent was obtained in written form.

### Reagents and Antibodies

SiRNA was purchased from Qiagen, dsiRNA were obtained from Integrated DNA Technologies, and the target sequences are listed in **S1 Table**. The following antibodies were used: monoclonal mouse anti-HA (MMS101R, Covance), monoclonal mouse anti-CFTR antibodies (10B6.2, 570 and 596, Cystic Fibrosis Foundation Therapeutics, Inc.), polyclonal rabbit anti-Rpl12 (AP16275c, Abgent), polyclonal rabbit anti-Rpl12 (ab157130, abcam), monoclonal rat anti-HSP90A (9D2, Enzo), monoclonal rat anti-HSPA8 (1B5, Enzo), mouse monoclonal anti-HSPA4 (C92F3A-5, Enzo), monoclonal mouse anti-AHA1 (1A2-A8, Abnova), monoclonal mouse anti-STIP1 (DS14F5, Enzo), polyclonal rabbit anti-DNAJA1 (ADI-SPA-405, Enzo), monoclonal mouse anti-BAG1 (4A2, Enzo), monoclonal mouse anti-Mucin5A/C (45M1, ThermoScientific), monoclonal mouse anti-acetylated tubulin (6-11B-1, Sigma), polyclonal rabbit anti-occludin (Zymed), monoclonal mouse anti-Na^+^/K^+^-ATPase (H3, Santa Cruz Biotechnology), monoclonal mouse anti-βactin (ab8226, abcam), and monoclonal mouse anti-βactin (AC-15, Sigma). The TfR PM density was detected using HRP-conjugated transferrin (090-030-050, Jackson ImmunoResearch), and actin was labeled for immunofluorescence microscopy using A555 conjugated Acti-stain (Cystoskeleton).

### Yeast Strains and Media

Yeast deletion mutant strains were obtained from Research Genetics. To retest selected hits, four single-colony deletion mutant clones were selected for construction of double mutants by the SGA, as described [44,45]. The query allele of the RL8 strain (described in [39]) was modified by substitution for the *Tet* promoter with the *ACT1* promoter (*Pact1*) and without the C-terminal GFP tag to obtain the *Pact1-yor1-F670-R1116T-HA* allele. To construct this allele, the pJ023 plasmid was modified by replacing the tetracycline promoter element with 680 bp of the 5’ UTR of *ACT1* promoter and then used to construct *Pact1-yor1-ΔF670-R1116T-HA* (the R1116T second site mutation is described in [36]) by integrating it at the *YOR1* promoter of LMY789, containing the *yor1-ΔF670-R1116T-HA* allele (gift from Elizabeth Miller). After SGA, frozen glycerol stocks were made for subsequent Q-HTCP analysis. YPEG media was made with 10 g/L yeast extract, 20 g/L peptone, 3% ethanol, and 3% glycerol.

### Q-HTCP

Q-HTCP was performed as previously described [46], assessing gene interaction by comparison of the cell proliferation parameter, L (the time (hours) after which half carrying capacity is reached), obtained from growth curves for single and double mutant cultures across a range of perturbation intensity using different growth-inhibitory concentrations of oligomycin, the primary substrate of Yor1. Hundreds of replicates of the single mutant, Yor1-ΔF670, were used to obtain the range of L at each condition tested. Interaction was calculated as the difference between the L values for the double mutant and the Yor1-ΔF670 single mutant, after normalizing all data by the difference in L between the double mutant and the Yor1-ΔF670 single mutant reference median in media not containing oligomycin, as previously described [39].

### Cell Culture and Media

Human lung tissues were obtained from *CFTR*^*WT/WT*^ non-CF and *CFTR*^*ΔF508/ΔF508*^ CF individuals (patient code 21, 22, and 48) under the protocol and consent form approved by the Institutional Review Board at University of Alabama Birmingham (IRB #X080625002). Cell isolation was performed as described [51,99]. After expansion, first or second passage cells were transferred to permeable filter supports and allowed to differentiate under air–liquid interface conditions for 3 wk as described in [100,101]. Alternatively, HBE cells (patient code BCFr43 and BCF121209) were purchased from the Cystic Fibrosis Translational Research center (CFTRc) at McGill University, expanded following the conditional reprogramming protocol described in [102] followed by differentiation on permeable filter supports under air–liquid interface using the Vertex conditions [100].

The generation and maintenance of the CFBE cell lines, expressing WT- or ΔF508-CFTR with a 3HA-tag in the 4th extracellular loop under the control of a tetracycline-inducible transactivator was described before [17,48]. Generation of stable CFBE cell lines constitutively expressing WT- or ΔF508-CFTR has been described and these cell lines are depicted as CFBE^C^ [51,52] to distinguish them from the inducible CFBE expression system. The HeLa cells constitutively expressing extracellular 3HA-tagged WT- or ΔF508-CFTR have been described previously [11].

### SiRNA Transfection

In CFBE cells, the target genes were silenced by forward transfection using 50 nM siRNA (Qiagen) and Lipofectamine RNAiMAX (Invitrogen). Transfected cells were allowed to polarize for 5 d. For studies in filter grown CFBE cells, 100 nM siRNA duplexes were introduced by reverse transfection using an established protocol [53]. HeLa cells were transfected using Oligofectamine (Invitrogen) as described previously [11], and the experiments were performed 4 d after transfection. HBE cells were either reverse transfected with dsiRNA (Integrated DNA Technologies) using Lipofectamine RNAiMAX [53] or were forward transfected for 6 h 1 d postseeding with 50 nM NT or RPL12_6 siRNA and the transfections were repeated every 7 d. Monolayers were cultured for a total of 21 d.

### PM Density Measurement

PM densities of extracellular HA-tagged proteins were determined by cell surface ELISA [11]. The TfR PM density was measured by the specific binding of HRP-conjugated transferrin. The relative amount of transferrin-HRP or HRP-conjugated secondary antibodies was measured by luminescence, using a VICTOR Light plate reader (PerkinElmer) after addition of 50 μl/well HRP-Substrate (SuperSignal West Pico, Thermo Fisher Scientific). PM density measurements were normalized for cell number, determined by Alamar Blue assay (Invitrogen) or protein concentration, determined by bicinchoninic acid assay (BCA, Pierce).

### CFTR or TMEM16A Conductance Measurement by Halide-Sensitive YFP Quenching Assay

The halide-sensitive YFP quenching assay has been used before to determine the PM function of CFTR [19] or TMEM16A [48]. Briefly, ΔF508-CFTR was activated by well-wise injection of 50 μl/well activator solution (20 μM Frk, 0.5 mM IBMX, 0.5 mM cpt-cAMP and 100 μM gen in PBS) followed by 100 μl PBS-iodide in which NaCl was replaced with NaI after a delay of 60 s. TMEM16A was activated by well-wise injection of 50 μl/well 100 μM ATP in PBS followed by 100 μl PBS-iodide after a delay of 6 s.

### Immunoblotting

For immunoblot analysis, cells were lysed either with NP-40-based lysis buffer (150 mM NaCl, 1% NP-40, 50 mM Tris-HCl, pH 8.0) or RIPA (ThermoScientific) in the presence of Halt protease inhibitor cocktail (ThermoScientific). Following total protein quantification using the BCA assay (ThermoScientific), equal amounts of protein were mixed with 4X loading buffer, incubated at 37°C for 10 min, resolved by SDS-PAGE, and blotted onto PVDF membranes. Following antibody binding, signals were detected using the SuperSignal West Femto (ThermoScientific) or Luminata Crescendo (EMD Millipore) substrates and quantified on a ChemiDocXRS (Bio-Rad) or by densitometry using ImageJ.

### Thermoaggregation Assay

Thermoaggregation assays were performed as previously described [66]. Briefly, following cell lysis with RIPA buffer, the lysates were cleared by centrifugation and the aggregation tendency of WT- and ΔF508-CFTR was compared after exposing the lysates to 20–80°C for 15 min. Macromolecular aggregates were eliminated by centrifugation (15,000 × g for 15 min). The remaining soluble WT- and ΔF508-CFTR, and HSPA4 in the supernatant was measured by quantitative immunoblotting.

### I_sc_ Measurement

CFBE or HBE cells expressing WT- or ΔF508-CFTR were grown to confluence on permeable filters at an air-liquid interface and mounted in modified Ussing chambers. I_sc_ measurements were obtained under voltage clamp conditions using MC8 equipment and P2300 Ussing chambers (Physiologic Instruments) as previously described [51,52,103]. Cells were equilibrated for 5–10 min in regular Ringer solution (115 mM NaCl, 25 mM NaHCO_3_, 2.4 mM KH_2_PO_4_, 1.24 mM K_2_HPO_4_, 1.2 mM CaCl_2_, 1.2 mM MgCl_2_, 10 mM D-glucose, pH 7.4). In some measurements, this was followed by the exchange of low chloride Ringer (115 mM NaCl reduced to 1.2 mM NaCl and 10 mM D-glucose replaced with 115 mM Na-gluconate) to the apical surface. After addition of the sodium channel inhibitor amiloride (100 μM), the CFTR agonists Frk (10 μM) and gen (50 μM) were sequentially added, followed by inhibitor_172_ (10 μM) at the conclusion of each experiment, in order to specifically inhibit CFTR activity. Changes in CFTR-mediated ion transport were calculated using the highest current value for each sample after achievement of a stable plateau for several minutes.

### Metabolic Pulse-Chase Studies

Experiments were performed essentially as described [104]. Briefly, 4–5 d after NT or *RPL12* siRNA transfection, CFBE (or HeLa) cells expressing ΔF508-CFTR were pulse-labeled with 0.2 mCi/ml (or 0.1 mCi/ml for HeLa cells) ^35^S-methinonine and ^35^S-cysteine (EasyTag Express Protein Labeling Mix, PerkinElmer) in cysteine and methionine-free medium for 30 min for CFBE (or 20 min for HeLa) at 26°C or 37°C (incorporation efficiency) or labeled for 3 h at 26°C and then chased 2 h at 37°C in full medium (maturation efficiency). Radioactivity incorporated into the core- and complex-glycosylated glycoproteins was visualized by fluorography and quantified by phosphoimage analysis using a Typhoon imaging platform (GE Healthcare). For depiction of representative images, autoradiographs were acquired by film-exposure.

For the experiments involving VX-809, NT, or *RPL12*, siRNA-transfected HeLa cells expressing ΔF508-CFTR were treated with 3 μM VX-809 or DMSO for 24 h before the experiment. Cells were pulse-labeled with 0.1 mCi/ml for 20 min and chased 2 h at 37°C in the presence or absence of VX-809. The maturation efficiency was determined by calculating the percent of pulse-labeled immature, core-glycosylated ΔF508-CFTR conversion into the mature, complex-glycosylated form. To allow for the detection of the low percentage of complex-glycosylated ΔF508-CFTR, the total labeling for 3 h was extrapolated from values obtained for 20 or 30 min pulse labeled samples.

### Ribosomal Run-Off Assay

HeLa cell transfected with either NT or RPL12_6 siRNA were treated with harringtonine (2 μg/ml) to stop translation initiation [69]. Ongoing elongation was quantified by the metabolic pulse chase technique, using ^35^S-methinonine and ^35^S-cysteine. The run-off elongation was terminated after 1–3 min with CHX (100 μg/ml). The cell lysates were separated by SDS-PAGE and the total radioactive signal of fluorographs was measured by phosphoimage analysis. The signals were normalized for the amount of protein loaded and expressed as percentage on non-CHX-treated controls.

### Q-PCR

Q-PCR was performed as described previously [48]. The primers are listed in **S2 Table**.

### Immunostaining and Fluorescence Microscopy

Filter-grown differentiated HBE cells were washed with PBS containing 5 mM DL-dithiothreitol for 5 min on ice to remove mucin. Cells were fixed in 4% paraformaldehyde and permeabilized with 0.2% Triton X-100. For occludin staining, cells were pre-extracted with 0.2% Triton X-100 for 2 min before fixation. After blocking in PBSCM (PBS containing 1 mM MgCl_2_, 0.1 mM CaCl_2_) with 0.5% bovine serum albumin, cells were incubated with primary antibodies overnight at 4°C, washed with PBSCM, and stained with Alexa-Fluor-conjugated secondary antibodies (1:1000) or Acti-stain (1:300) for 1 h at room temperature. Nuclei were stained with DAPI. Cut-out pieces of filter were mounted on glass slides, and optical stacks were acquired using a laser confocal fluorescence microscope (LSM780 Carl Zeiss) equipped with a 63X/1.40 oil DICIII Plan apochromat objective. Typically 20–30 optical xy-sections were acquired and reconstituted using the Zen 2012 software, and representative xz-sections are shown.

### Statistical Analysis

Results are presented as mean ± SEM with the number of experiments indicated. Statistical analysis was performed by two-tailed Student's *t* test with the means of at least three independent experiments, and the 95% confidence interval was considered significant.

## Supporting Information

S1 Data Numerical values used in preparation of Figs 1A–1E, 2A, 2C, 2E, 2F, 3A–3E, 4A–4C, 5B–5D, 6A–6F, 7B, 7D–7H, 8B–8D, 9A–9D, S1B, S2C–S2E, S3B–S3E, S4C, S5A–S5C, S5E, S5F, S6A–S6C, S7A–S7E and S7H–S7L. (XLS)

S1 Fig Analysis of hits from genome-wide screen for modifiers of Yor1-ΔF670 biogenesis. **(A)** The top 180 deletion suppressors [39] were reanalyzed using the DAVID bioinformatics tool and functional annotation clustering. Two clusters were identified with an enrichment score of 5.68 (left panel) and 4.78 (right panel). Only genes with more than three associations to gene ontology (GO) terms, SwissProt—Protein Information Resource (SP_PIR) keywords or Kyoto Encyclopedia of Genes and Genomes (KEGG) pathways are shown. Genes selected for further study are highlighted in red. **(B)** Scatter plot of the change in the cell proliferation parameter L (ΔL) for 0.25 μg/ml oligomycin in *yor1-ΔF670*. Oligomycin resistance was compared between the single mutant (control) and the indicated double mutant cultures. The same results depicted as box-whisker plots are shown in Fig 1B–1E. **p < 0.01; ***p < 0.001. The underlying data of panel B can be found in S1 Data.
(TIF)

S2 Fig Halide-sensitive YFP quenching assay in CFBE cells expressing ΔF508-CFTR. **(A, B)** Representative traces of rΔF508-CFTR function assayed by halide-sensitive YFP quenching in CFBE cells in combination with knockdown with two individual siRNAs per indicated gene after 0 h (A) or 3 h (B) chase at 37°C. CFBE cells expressing inducible ΔF508-CFTR and constitutive halide-sensitive YFP-F46L/H148Q/I152L were transfected with siRNA. The ΔF508-CFTR function was measured by determining the YFP quenching kinetics in response to extracellular iodide addition in the presence of Frk (10 μM), IBMX (250 μM), cpt-cAMP (250 μM) and gen (50 μM). **(C)** Correlation between the rΔ508-CFTR function as depicted in Fig 2C and the TMEM16A function monitored by iodide-mediated YFP quenching in CFBE in combination with knockdown of the Yor1-ΔF670 modifier homologs (*n* = 2). **(D)** Correlation between the rΔF508-CFTR PM density as depicted in Fig 2A and TfR PM density determined by transferrin-HRP binding (*n* = 3). **(E)** Correlation between the rΔF508-CFTR PM density as depicted in Fig 2A and MLC1-S280L PM density determined by cell surface ELISA (*n* = 3). Error bars indicate SEM of 2–6 independent experiments. The underlying data of panels C–E can be found in S1 Data.
(TIF)

S3 Fig The effect of siRNA-mediated *RPL12* knockdown on the PM density and function of ΔF508-CFTR. **(A, B)** Knockdown efficiency of *RPL12* by two individual siRNAs was determined in polarized CFBE after 5 days of transfection by immunoblotting (A) or qPCR (B, *n* = 3). **(C)** Effect of *RPL12* knockdown on the PM density of rΔF508-CFTR in HeLa cells (*n* = 5). **(D, E)** Representative I_sc_ recordings (upper panel) and quantification of the changes (ΔI_sc_, lower panel) after siRNA-mediated *RPL12* knockdown, NT siRNA or mock transfection in CFBE cell monolayers expressing WT CFTR (D, *n* = 5), or HBE cells homozygous for WT CFTR from one donor (E, *n* = 3, donor code 10). CFTR-mediated currents were induced by sequential addition of Frk (10 μM) and gen (50 μM) followed by CFTR inhibition with inhibitor_172_ (10 μM) in the presence of a basolateral-to-apical chloride gradient. **p < 0.01; ***p < 0.001. Error bars indicate SEM of 3–5 independent experiments. The underlying data of panels B–E can be found in S1 Data.
(TIF)

S4 Fig *RPL12* silencing in HBE, alone or in combination with VX-809, increases the ΔF508-CFTR function but does not affect differentiation or morphology of the cells. **(A)** Knockdown efficiency of *RPL12* by two individual dsiRNAs in polarized HBE 21 d after transfection determined by immunoblot. **(B, C)** Representative I_sc_ recordings (B) and quantification of the changes in I_sc_ upon CFTR inhibition with Inh_172_ (ΔI_sc_ Inh_172_, C) after dsiRNA-mediated *RPL12* knockdown or NT dsiRNA transfection in HBE cells homozygous for ΔF508-CFTR CFTR (patient codes BCFr34 and BCF121209, *n* = 3). CFTR mediated currents were induced by sequential addition of Frk (20 μM) and gen (50 μM) followed by CFTR inhibition with Inh_172_ (20 μM) in the presence of equimolar chloride concentrations in both chambers. **(D, E)** Characterization of HBE cells in the presence of *RPL12* silencing. Primary HBE from two patients with *CFTR*^*ΔF508/ΔF508*^ genotype (D–BCFr43, E–BCF121209) were transfected with control (NT) or *RPL12* (RPL12_6 and _12) dsiRNAs and differentiated for 3 wk at air–liquid interface. The cells were fixed, permeabilized, and differentiation of the pseudostratified epithelial layer was verified by the presence of ciliated cells (acetylated tubulin, Ac.tub.), goblet cells (mucin5A/C) and the staining pattern of occluding, a tight-junctions marker. DAPI was used to stain nuclei. Dotted lines show the filter, Ap, apical, scale bar = 10 μm. The transepithelial resistance, an indirect marker of the integrity of the monolayer was also preserved (NT 403: ± 50 Ω/cm^2^, RPL12_6: 351 ± 27 Ω/cm^2^, RPL12_11: 384 ± 45 Ω/cm^2^). Error bars show SEM of three independent experiments. The underlying data of panel C can be found in S1 Data.
(TIF)

S5 Fig *RPL12* silencing does not increase ΔF508-CFTR mRNA expression, but augments the fractional Frk stimulated current. **(A)** Relative amount of CFTR mRNA in CFBE cells upon transfection with *RPL12* or NT siRNAs determined by qPCR (*n* = 3). **(B)** Cell number determined by Alamar blue assay (*n* = 3) or protein concentration measured by BCA assay (*n* = 3) of CFBE upon *RPL12* knockdown for 5 d in comparison to NT siRNA or 24 h treatment with VX-809 (3 μM). (**C**) Determination of [^35^S]-methionine/cysteine incorporation during the labeling period into the nascent ΔF508-CFTR (pulse 10 min) at 37°C in RPL12 or NT siRNA treated HeLa cells (*n* = 3). **(D)** Following *RPL12* knockdown at 37°C in CFBE^C^ cells, the half-life of ΔF508-CFTR was determined by immunoblot with CHX chase. Visualization of CFTR was achieved using 10B6.2 antibody, and anti-β-actin antibody was utilized as a loading control. (**E, F**) Fractional Frk-stimulated activity of ΔF508-CFTR in CFBE^C^ (E, *n* = 5) or HBE (F, *n* = 4) with *CFTR*^*ΔF508/ΔF508*^ genotype. The relative Frk sensitivity was calculated as a ratio of I_sc_ stimulated with Frk (10 μM) over Frk and gen (50 μM) as shown in Figs 3E and 4B. *p < 0.05, **p < 0.01; ***p < 0.001. Error bars show SEM of 3–5 independent experiments. The underlying data of panels A–C, E, and F can be found in S1 Data.
(TIF)

S6 Fig *RPL12* silencing effect on the stability of misfolded PM proteins in HeLa cells. **(A–C)** PM stability determined by cell surface ELISA in NT or *RPL12* siRNA treated HeLa cells. Cells stably expressing extracellular HA-epitope tagged V2R-Y128S (A, *n* = 3), MLC1-P92S or -S280L (B, *n* = 3), or hERG-WT or -G601S (C, *n* = 4) were used. SiRNAs for CHIP and Tsg101 served as positive controls that attenuated the peripheral removal of misfolded membrane proteins. The amount remaining after initial labeling is calculated as percent after the indicated chase time. *p < 0.05; **p < 0.01; ***p < 0.001. Error bars are SEM of 3–4 independent experiments. The underlying data of panels A–C can be found in S1 Data.
(TIF)

S7 Fig Ribosomal stalk protein, eEF-2 and eIF4E knockdown in CFBE cells. **(A–E)** Knockdown efficiency of RPLP0 (A), RPLP1 (B), RPLP2 (C), eEF-2 (D), and eIF4E (E) in polarized CFBE 5 d after transfection as determined by qPCR (*n* = 3). (**F, G**) Correlation between the knockdown efficiency of RPLP0, RPLP1, RPLP2 or eEF-2 and the rΔF508-CFTR PM density (F) or function (G). **(H, J)** The effect of the core initiation factor eIF4E knockdown on the PM density (H) and function (J) of rΔF508-CFTR in CFBE. The values are expressed as percent of NT siRNA controls (*n* = 3). **(I, K)** The effect of eIF4E silencing on the PM (I, *n* = 3) and functional stability (K, *n* = 3) of rΔF508-CFTR after 1.5 and 3 h chase at 37°C. (**L**) [^35^S]-methionine and [^35^S]-cysteine incorporation during the labeling period (15 min) at 37°C into the newly formed ΔF508-CFTR in CFBE cells transfected with EIF4E or NT siRNA (*n* = 4). *p < 0.05; **p < 0.01; ***p < 0.001. Error bars show SEM of 3–6 independent experiments. The underlying data of panels A–E and H–L can be found in S1 Data.
(TIF)

S8 Fig The effect of *RPL12* knockdown on CFTR-associated chaperones. Expression of the CFTR-associated chaperones and cochaperones HSP90A, HSPA8, Aha1, STIP1, DNAJA1 and BAG1 was determined by immunoblot analysis upon knockdown of *RPL12*. β-actin and Na^+^/K^+^-ATPase served as loading controls.
(TIF)

S1 Table SiRNAs used in this study. (PDF)

S2 Table Q-PCR primers used in this study. (PDF)

## Abbreviations

ABC: ATP-binding cassette
ABCC: ATP-binding cassette family C
BCA: bicinchoninic acid
CF: cystic fibrosis
CFTR: cystic fibrosis transmembrane conductance regulator
CFTRc: Cystic Fibrosis Translational Research center
CHX: cycloheximide
cpt-cAMP: 8-(4-Chlorophenylthio)-adenosine-3',5'-cyclic monophosphate
Frk: forskolin
FRT: Fisher rat thyroid
GAC: GTPase-associated center
gen: genistein
GO: gene ontology
hERG: human Ether-à-go-go-Related Gene
IBMX: 3-Isobutyl-1-methyl-xanthine
Inh_172_: inhibitor 172
I_sc_: short-circuit current
KEGG: Kyoto Encyclopedia of Genes and Genomes
MLC1: megalencephalic leukoencephalopathy with subcortical cyst 1
NBD1: nucleotide binding domain 1
NT: nontargeted
PKA: protein kinase A
PM: plasma membrane
Q-HTCP: quantitative high throughput cell array phenotyping
rΔF508: low-temperature rescued ΔF508-CFTR
SGA: synthetic genetic array
SP_PIR: SwissProt—Protein Information Resource
TfR: transferrin receptor
V2R: vasopressin 2 receptor
YOR1: yeast oligomycin resistance 1
