# Correction: Ribosomal Stalk Protein Silencing Partially Corrects the ΔF508-CFTR Functional Expression Defect

**DOI:** 10.1371/journal.pbio.1002574

**Published:** 2016-11-02

**Authors:** The PLOS Biology Staff

## Notice of Republication

This article was republished on October 11th, 2016, to correct a technical error in the author byline in the online version of this article. The suffix “IV” for author John L. Hartman IV was incorrectly tagged as part of his surname.
