# Supplementary figures and images for: Ribosomal Stalk Protein Silencing Partially Corrects the ΔF508-CFTR Functional Expression Defect
Source: PLoS Biol. 2016 May 11;14(5):e1002462. doi: 10.1371/journal.pbio.1002462 (PMC4864299; doi:10.1371/journal.pbio.1002462)

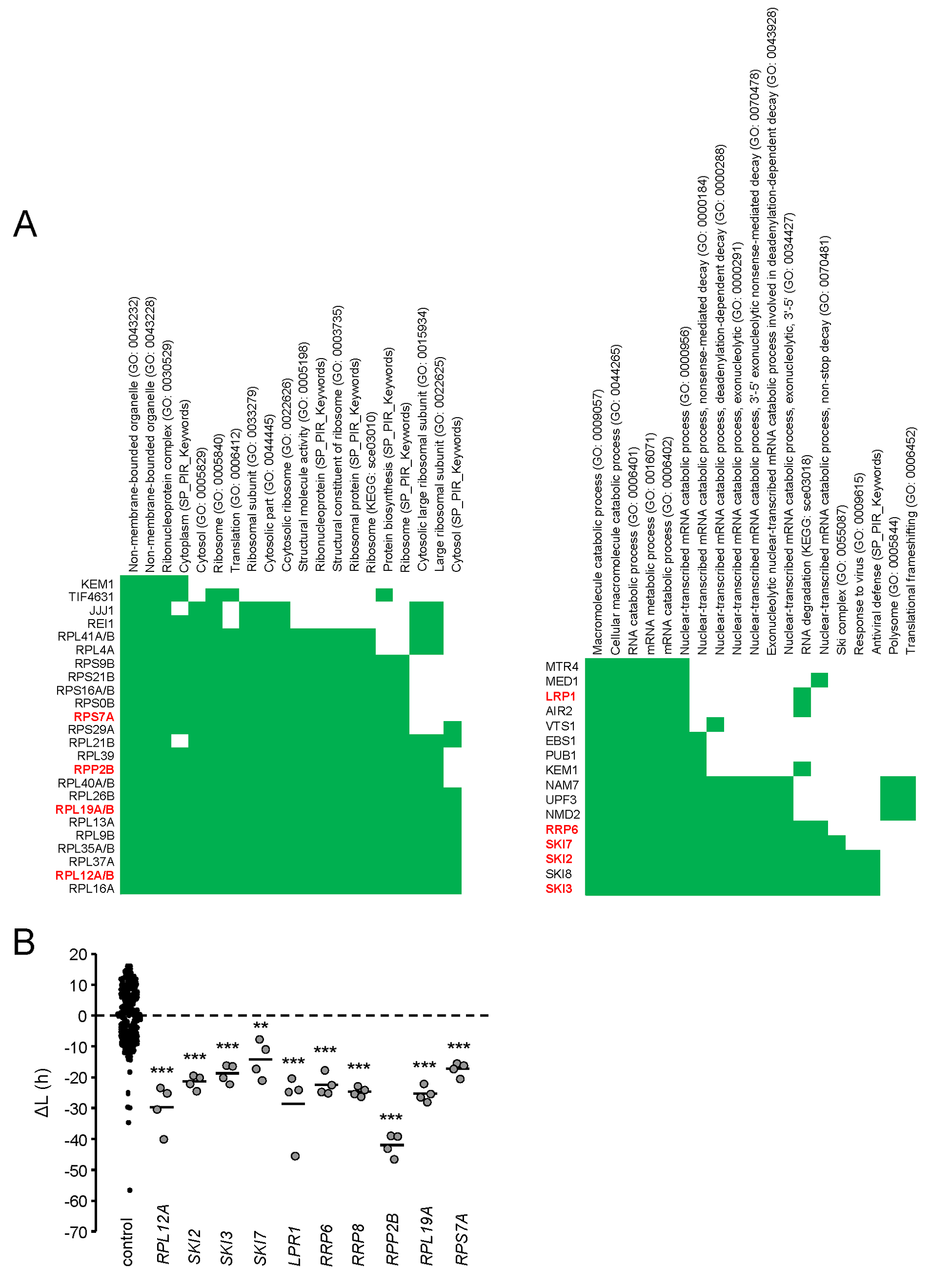

Supplement: S1 Fig — (A) The top 180 deletion suppressors [39] were reanalyzed using the DAVID bioinformatics tool and functional annotation clustering. Two clusters were identified with an enrichment score of 5.68 (left panel) and 4.78 (right panel). Only genes with more than three associations to gene ontology (GO) terms, SwissProt—Protein Information Resource (SP_PIR) keywords or Kyoto Encyclopedia of Genes and Genomes (KEGG) pathways are shown. Genes selected for further study are highlighted in red. (B) Scatter plot of the change in the cell proliferation parameter L (ΔL) for 0.25 μg/ml oligomycin in yor1-ΔF670. Oligomycin resistance was compared between the single mutant (control) and the indicated double mutant cultures. The same results depicted as box-whisker plots are shown in Fig 1B–1E. **p < 0.01; ***p < 0.001. The underlying data of panel B can be found in S1 Data. (TIF) [file pbio.1002462.s002.tif]

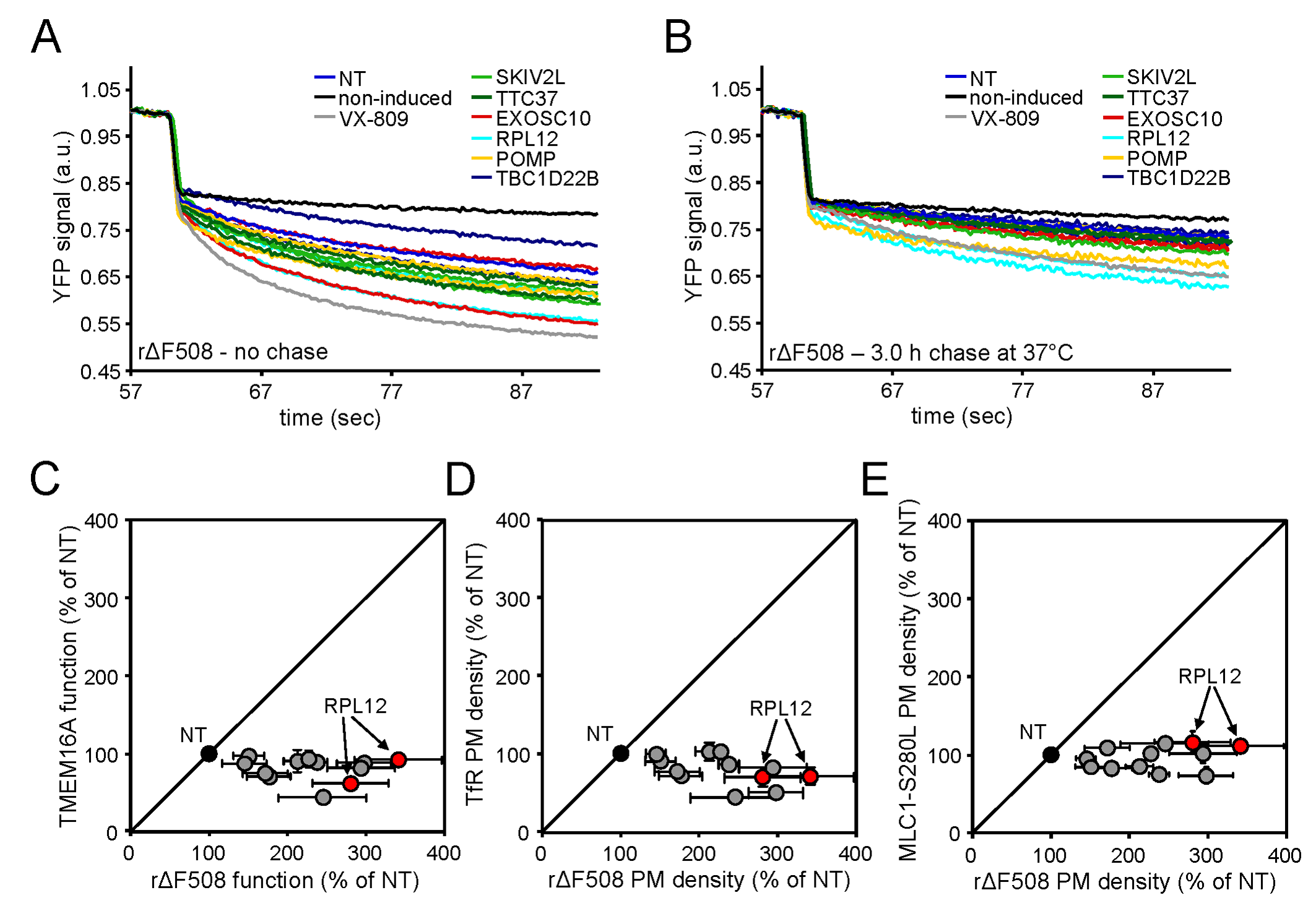

Supplement: S2 Fig — (A, B) Representative traces of rΔF508-CFTR function assayed by halide-sensitive YFP quenching in CFBE cells in combination with knockdown with two individual siRNAs per indicated gene after 0 h (A) or 3 h (B) chase at 37°C. CFBE cells expressing inducible ΔF508-CFTR and constitutive halide-sensitive YFP-F46L/H148Q/I152L were transfected with siRNA. The ΔF508-CFTR function was measured by determining the YFP quenching kinetics in response to extracellular iodide addition in the presence of Frk (10 μM), IBMX (250 μM), cpt-cAMP (250 μM) and gen (50 μM). (C) Correlation between the rΔ508-CFTR function as depicted in Fig 2C and the TMEM16A function monitored by iodide-mediated YFP quenching in CFBE in combination with knockdown of the Yor1-ΔF670 modifier homologs (n = 2). (D) Correlation between the rΔF508-CFTR PM density as depicted in Fig 2A and TfR PM density determined by transferrin-HRP binding (n = 3). (E) Correlation between the rΔF508-CFTR PM density as depicted in Fig 2A and MLC1-S280L PM density determined by cell surface ELISA (n = 3). Error bars indicate SEM of 2–6 independent experiments. The underlying data of panels C–E can be found in S1 Data. (TIF) [file pbio.1002462.s003.tif]

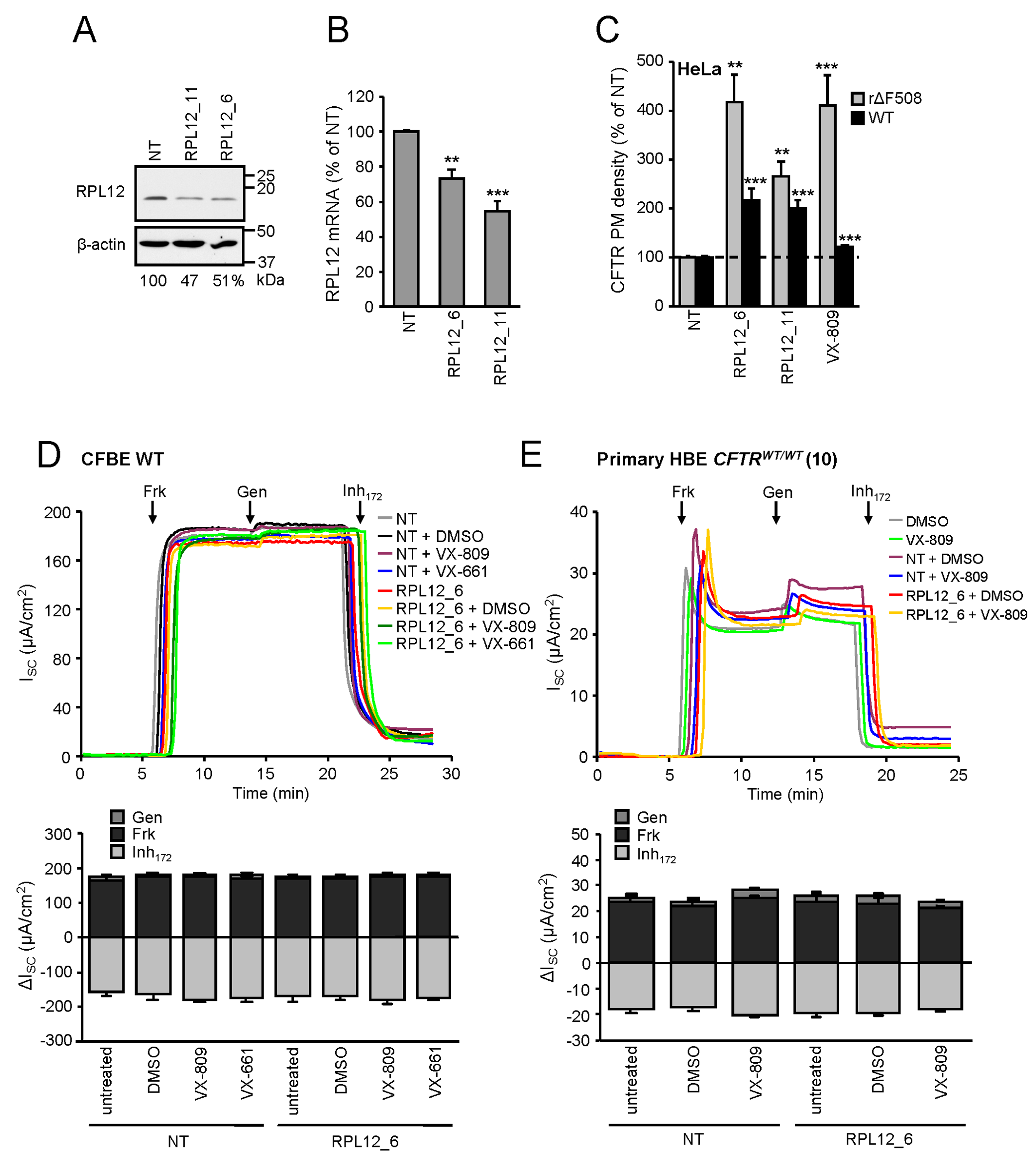

Supplement: S3 Fig — (A, B) Knockdown efficiency of RPL12 by two individual siRNAs was determined in polarized CFBE after 5 days of transfection by immunoblotting (A) or qPCR (B, n = 3). (C) Effect of RPL12 knockdown on the PM density of rΔF508-CFTR in HeLa cells (n = 5). (D, E) Representative Isc recordings (upper panel) and quantification of the changes (ΔIsc, lower panel) after siRNA-mediated RPL12 knockdown, NT siRNA or mock transfection in CFBE cell monolayers expressing WT CFTR (D, n = 5), or HBE cells homozygous for WT CFTR from one donor (E, n = 3, donor code 10). CFTR-mediated currents were induced by sequential addition of Frk (10 μM) and gen (50 μM) followed by CFTR inhibition with inhibitor172 (10 μM) in the presence of a basolateral-to-apical chloride gradient. **p < 0.01; ***p < 0.001. Error bars indicate SEM of 3–5 independent experiments. The underlying data of panels B–E can be found in S1 Data. (TIF) [file pbio.1002462.s004.tif]

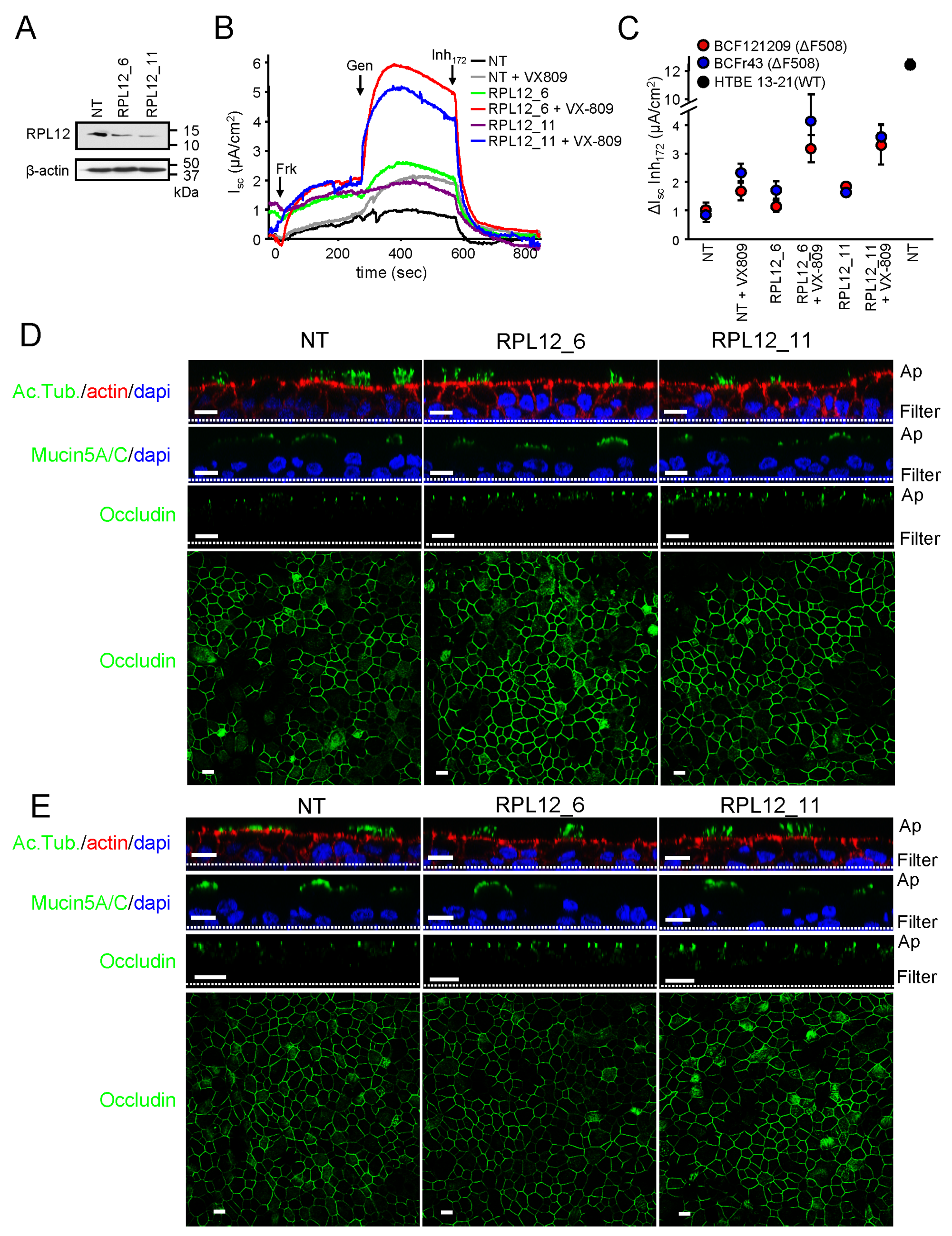

Supplement: S4 Fig — (A) Knockdown efficiency of RPL12 by two individual dsiRNAs in polarized HBE 21 d after transfection determined by immunoblot. (B, C) Representative Isc recordings (B) and quantification of the changes in Isc upon CFTR inhibition with Inh172 (ΔIsc Inh172, C) after dsiRNA-mediated RPL12 knockdown or NT dsiRNA transfection in HBE cells homozygous for ΔF508-CFTR CFTR (patient codes BCFr34 and BCF121209, n = 3). CFTR mediated currents were induced by sequential addition of Frk (20 μM) and gen (50 μM) followed by CFTR inhibition with Inh172 (20 μM) in the presence of equimolar chloride concentrations in both chambers. (D, E) Characterization of HBE cells in the presence of RPL12 silencing. Primary HBE from two patients with CFTRΔF508/ΔF508 genotype (D–BCFr43, E–BCF121209) were transfected with control (NT) or RPL12 (RPL12_6 and _12) dsiRNAs and differentiated for 3 wk at air–liquid interface. The cells were fixed, permeabilized, and differentiation of the pseudostratified epithelial layer was verified by the presence of ciliated cells (acetylated tubulin, Ac.tub.), goblet cells (mucin5A/C) and the staining pattern of occluding, a tight-junctions marker. DAPI was used to stain nuclei. Dotted lines show the filter, Ap, apical, scale bar = 10 μm. The transepithelial resistance, an indirect marker of the integrity of the monolayer was also preserved (NT 403: ± 50 Ω/cm2, RPL12_6: 351 ± 27 Ω/cm2, RPL12_11: 384 ± 45 Ω/cm2). Error bars show SEM of three independent experiments. The underlying data of panel C can be found in S1 Data. (TIF) [file pbio.1002462.s005.tif]

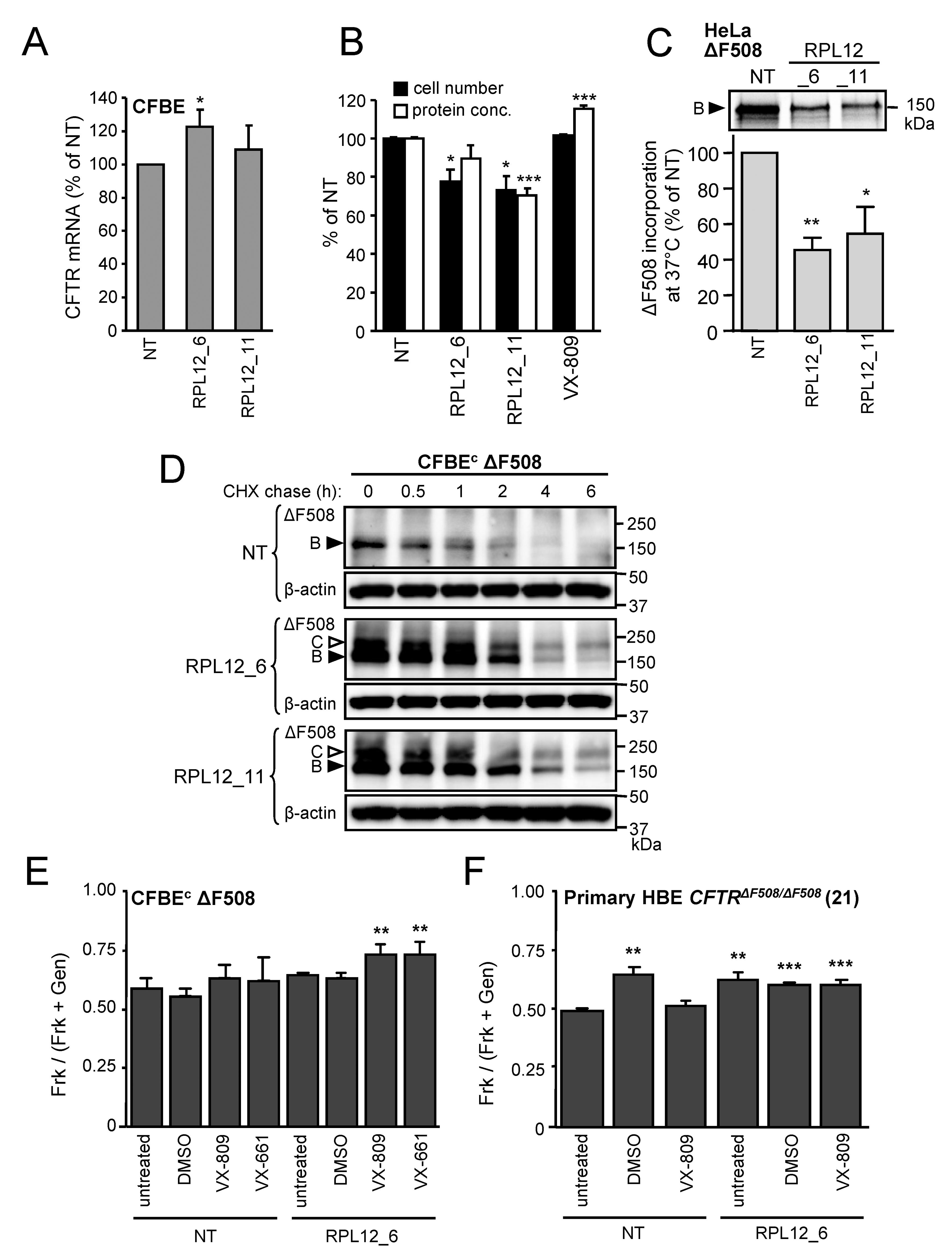

Supplement: S5 Fig — (A) Relative amount of CFTR mRNA in CFBE cells upon transfection with RPL12 or NT siRNAs determined by qPCR (n = 3). (B) Cell number determined by Alamar blue assay (n = 3) or protein concentration measured by BCA assay (n = 3) of CFBE upon RPL12 knockdown for 5 d in comparison to NT siRNA or 24 h treatment with VX-809 (3 μM). (C) Determination of [35S]-methionine/cysteine incorporation during the labeling period into the nascent ΔF508-CFTR (pulse 10 min) at 37°C in RPL12 or NT siRNA treated HeLa cells (n = 3). (D) Following RPL12 knockdown at 37°C in CFBEC cells, the half-life of ΔF508-CFTR was determined by immunoblot with CHX chase. Visualization of CFTR was achieved using 10B6.2 antibody, and anti-β-actin antibody was utilized as a loading control. (E, F) Fractional Frk-stimulated activity of ΔF508-CFTR in CFBEC (E, n = 5) or HBE (F, n = 4) with CFTRΔF508/ΔF508 genotype. The relative Frk sensitivity was calculated as a ratio of Isc stimulated with Frk (10 μM) over Frk and gen (50 μM) as shown in Figs 3E and 4B. *p < 0.05, **p < 0.01; ***p < 0.001. Error bars show SEM of 3–5 independent experiments. The underlying data of panels A–C, E, and F can be found in S1 Data. (TIF) [file pbio.1002462.s006.tif]

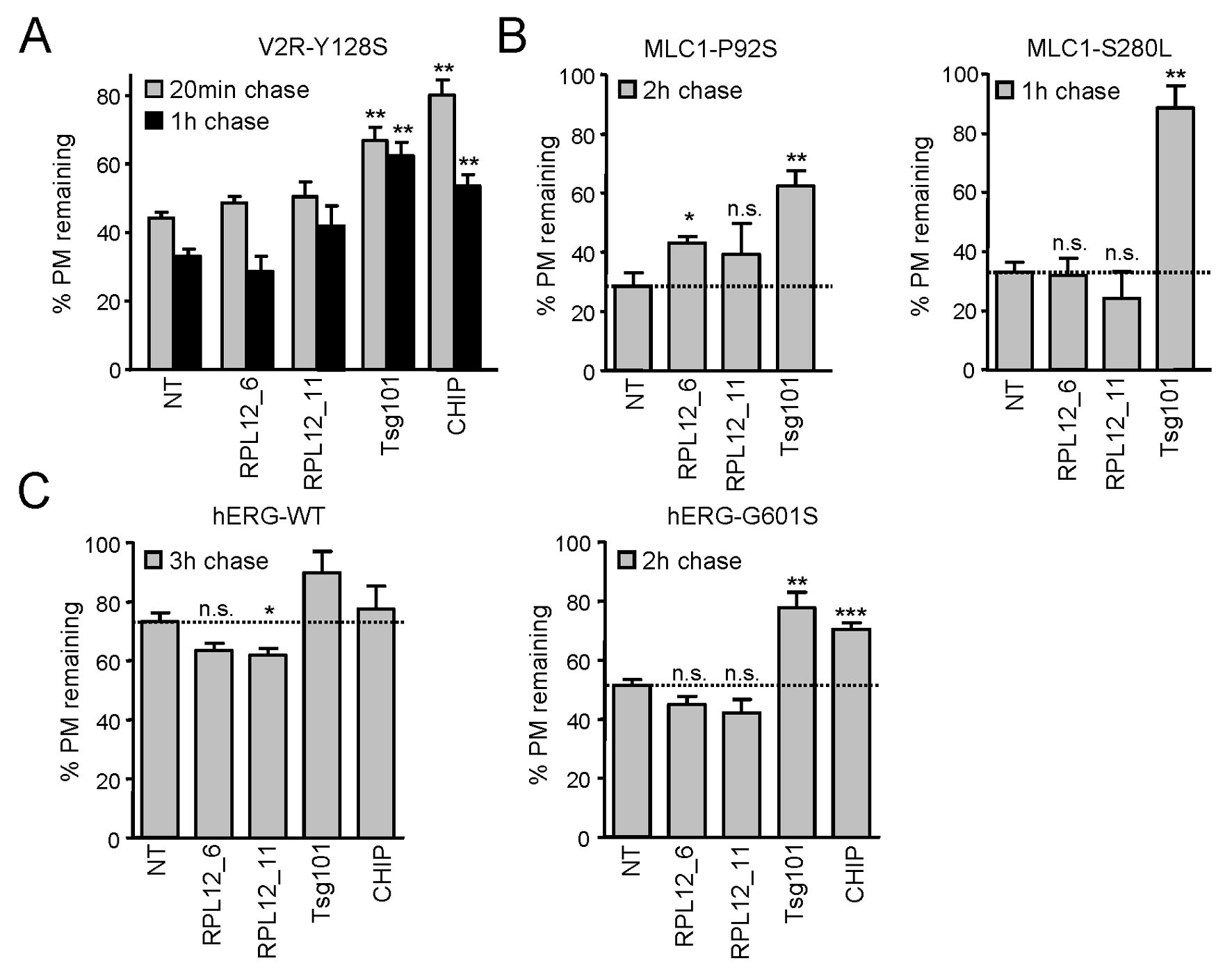

Supplement: S6 Fig — (A–C) PM stability determined by cell surface ELISA in NT or RPL12 siRNA treated HeLa cells. Cells stably expressing extracellular HA-epitope tagged V2R-Y128S (A, n = 3), MLC1-P92S or -S280L (B, n = 3), or hERG-WT or -G601S (C, n = 4) were used. SiRNAs for CHIP and Tsg101 served as positive controls that attenuated the peripheral removal of misfolded membrane proteins. The amount remaining after initial labeling is calculated as percent after the indicated chase time. *p < 0.05; **p < 0.01; ***p < 0.001. Error bars are SEM of 3–4 independent experiments. The underlying data of panels A–C can be found in S1 Data. (TIF) [file pbio.1002462.s007.tif]

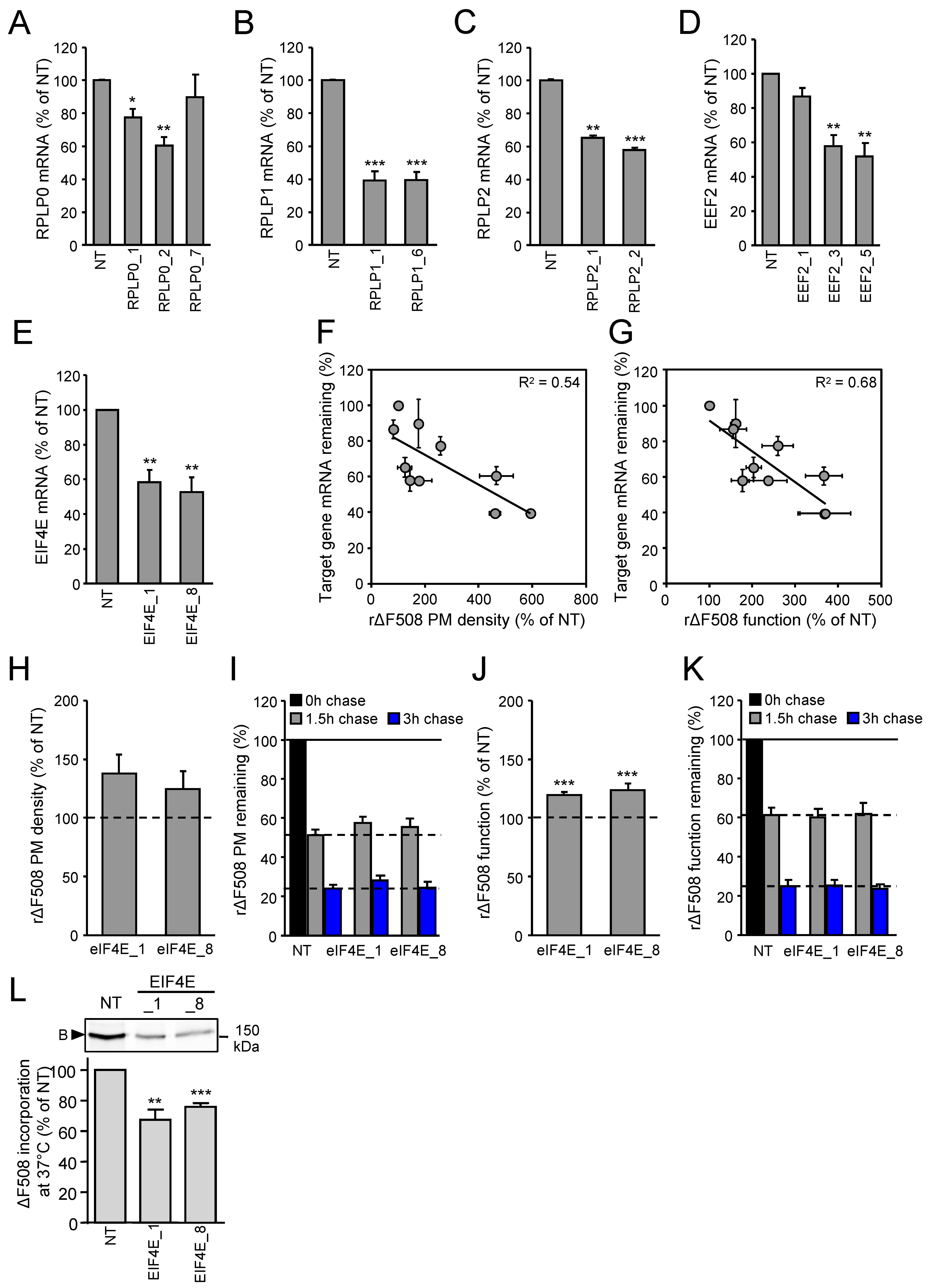

Supplement: S7 Fig — (A–E) Knockdown efficiency of RPLP0 (A), RPLP1 (B), RPLP2 (C), eEF-2 (D), and eIF4E (E) in polarized CFBE 5 d after transfection as determined by qPCR (n = 3). (F, G) Correlation between the knockdown efficiency of RPLP0, RPLP1, RPLP2 or eEF-2 and the rΔF508-CFTR PM density (F) or function (G). (H, J) The effect of the core initiation factor eIF4E knockdown on the PM density (H) and function (J) of rΔF508-CFTR in CFBE. The values are expressed as percent of NT siRNA controls (n = 3). (I, K) The effect of eIF4E silencing on the PM (I, n = 3) and functional stability (K, n = 3) of rΔF508-CFTR after 1.5 and 3 h chase at 37°C. (L) [35S]-methionine and [35S]-cysteine incorporation during the labeling period (15 min) at 37°C into the newly formed ΔF508-CFTR in CFBE cells transfected with EIF4E or NT siRNA (n = 4). *p < 0.05; **p < 0.01; ***p < 0.001. Error bars show SEM of 3–6 independent experiments. The underlying data of panels A–E and H–L can be found in S1 Data. (TIF) [file pbio.1002462.s008.tif]

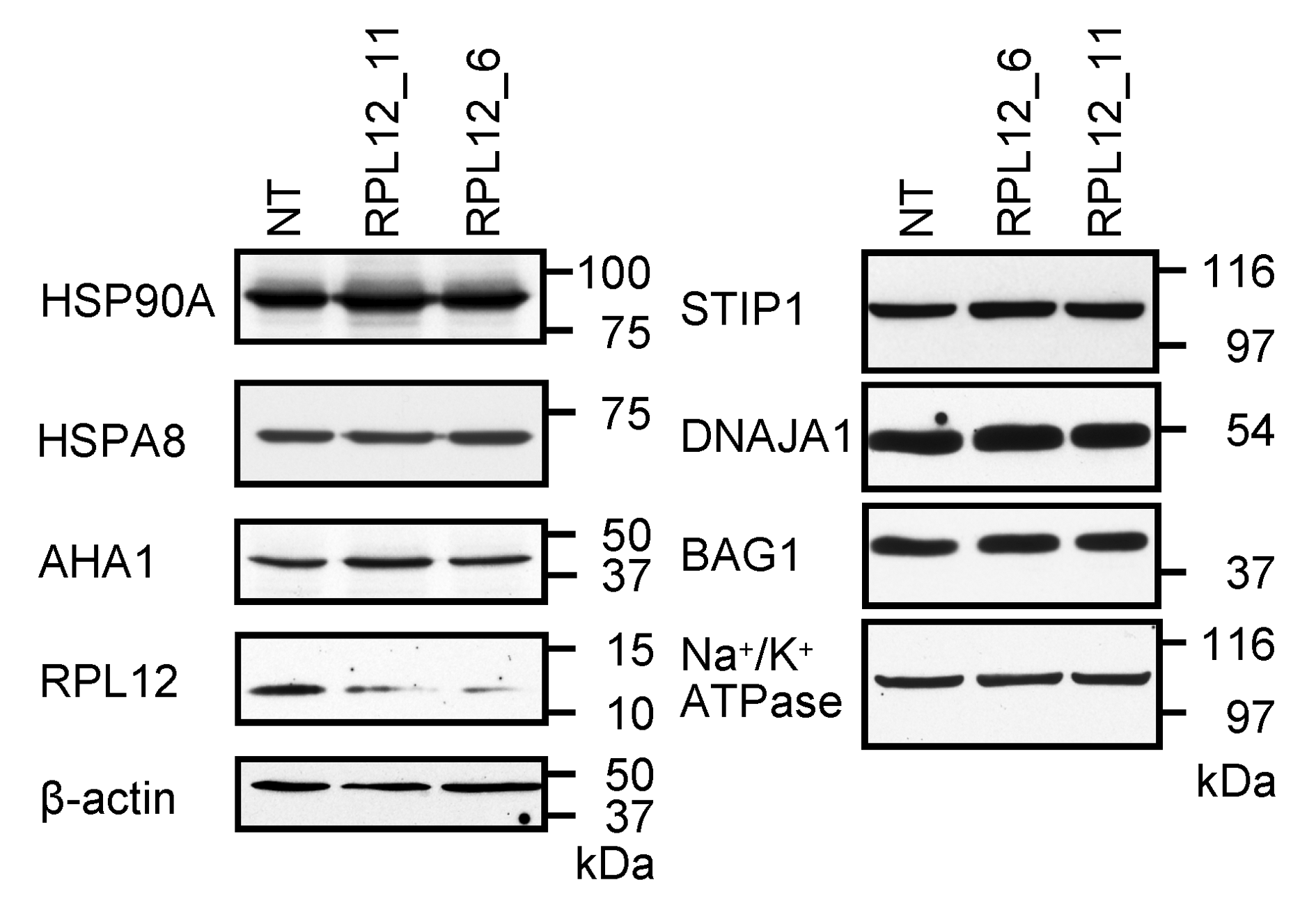

Supplement: S8 Fig — Expression of the CFTR-associated chaperones and cochaperones HSP90A, HSPA8, Aha1, STIP1, DNAJA1 and BAG1 was determined by immunoblot analysis upon knockdown of RPL12. β-actin and Na+/K+-ATPase served as loading controls. (TIF) [file pbio.1002462.s009.tif]
