# Supplementary material for: Ribosomal Stalk Protein Silencing Partially Corrects the ΔF508-CFTR Functional Expression Defect
Source: PLoS Biol. 2016 May 11;14(5):e1002462. doi: 10.1371/journal.pbio.1002462 (PMC4864299; doi:10.1371/journal.pbio.1002462)
Supplement: S1 Table — (PDF) [file pbio.1002462.s010.pdf]

**Gene symbol**	**Product name**	**SiRNA target sequence**
TBC1D22A	Hs_TBC1D22A_1	TACGATTCTAGGAACGACGAA
TBC1D22A	Hs_TBC1D22A_3	TACGTTCAGGGTATAAATGAT
TBC1D22B	Hs_TBC1D22B_5	CAGAACGGTCCCAGTCAACGA
TBC1D22B	Hs_TBC1D22B_7	CCAGGGAGGTTCGACCTATAA
RPL12	Hs_RPL12_6	CAGCCAGTTAAGCACAAAGGA
RPL12	Hs_RPL12_11	AACCACCAAGAGACAGAAAGA
RPSA	Hs_LAMR1_1	TCGACATGAGTTGTACTTCTA
RPSA	Hs_LAMR1_2	TACCTGGGATTGCATATCAAA
EXOSC10	Hs_EXOSC10_4	CAGGGACATCTGCCTCAAGAA
EXOSC10	Hs_EXOSC10_5	AGCCATCGTTAAGGTCTTTCA
EXOSC10	Hs_EXOSC10_6	AAGGAACCTCAGGGCATCATA
SKIV2L	Hs_SKIV2L_1	CACGTACACTATGATCCTCAA
SKIV2L	Hs_SKIV2L_2	CACCGTTATCCTGCTCTGCAA
SKIV2L	Hs_SKIV2L_4	CCCGAGGAGCCTTCCATACAA
POMP	Hs_POMP_1	CAGGTTCAGCGTCTTCCATTT
POMP	Hs_POMP_2	TTGGTCTGGTCTTTAAGTGAA
POMP	Hs_POMP_3	TAAACTTGGTTTACTGTAATA
TTC37	Hs_TTC37_5	TTGGGTTACTTAAACGAACAT
TTC37	Hs_TTC37_6	AAGCCGTAGACTACATAGAAA
RPLP0	Hs_RPLP0_1	AAGTGCTTGATATCACAGAGG
RPLP0	Hs_RPLP0_2	AATCCTGAGTGATGTGCAGCT
RPLP0	Hs_RPLP0_7	TTGGCTACTTTGTTCGCATTA
RPLP1	Hs_RPLP1_1	AAGAAAGTGGAAGCAAAGAAA
RPLP1	Hs_RPLP1_6	CTGCACGACGATGAGGTGACA
RPLP2	Hs_RPLP2_1	CAAGGTTATCAGTGAGCTGAA
RPLP2	Hs_RPLP2_2	AAGGAGGAGTCTGAAGAGTCA
EEF2	Hs_EEF2_1	CCGCGCCATCATGGACAAGAA
EEF2	Hs_EEF2_3	CTGGCCGAGGACATCGATAAA
EEF2	Hs_EEF2_5	TACCGCGAGACGGTCAGTGAA
EIF4E	Hs_EIF4E_1	AAGAGCGGCTCCACCACTAAA
EIF4E	Hs_EIF4E_8	CAAAGCTTTGCTACAAATTTA
