# Supplementary material for: Ribosomal Stalk Protein Silencing Partially Corrects the ΔF508-CFTR Functional Expression Defect
Source: PLoS Biol. 2016 May 11;14(5):e1002462. doi: 10.1371/journal.pbio.1002462 (PMC4864299; doi:10.1371/journal.pbio.1002462)
Supplement: S2 Table — (PDF) [file pbio.1002462.s011.pdf]

Gene symbol	Direction	Sequence
CFTR	forward	AGTGGAGGAAAGCCTTTGGAGT
CFTR	reverse	ACAGATCTGAGCCCAACCTCA
RPL12	forward	GACCCCAACGAGATCAAAGT
RPL12	reverse	TGGCAATGTCATCACCAACT
RPLP0	forward	GCGACCTGGAAGTCCAACTA
RPLP0	reverse	GGATCTGCTGCATCTGCTTG
RPLP1	forward	ACGGAGGATAAGATCAATGCC
RPLP1	reverse	CAGATGAGGCTCCCAATGTT
RPLP2	forward	ATCTTGGACAGCGTGGGTAT
RPLP2	reverse	ACCAGCAGGTACACTGGCA
EEF2	forward	CTGGAGATCTGCCTGAAGGA
EEF2	reverse	GACTTGGAGAGGCAGAGCAC
EIF4E	forward	GCCAATCCGGTTTGAATCT
EIF4E	reverse	GGGATTAGGAGTAGGGGTGG
GAPDH	forward	CATGAGAAGTATGACAACAGCCT
GAPDH	reverse	AGTCCTTCCACGATACCAAAGT
